# From Structure to Function: Understanding Synthetic Conditions in Relation to Magnetic Properties of Hybrid Pd/Fe-Oxide Nanoparticles

**DOI:** 10.3390/nano12203649

**Published:** 2022-10-18

**Authors:** Alexandra Maier, Rogier van Oossanen, Gerard C. van Rhoon, Jean-Philippe Pignol, Iulian Dugulan, Antonia G. Denkova, Kristina Djanashvili

**Affiliations:** 1 Department of Biotechnology, Delft University of Technology, Van Der Maasweg 9, 2629 HZ Delft, The Netherlands; 2 Department of Radiotherapy, Erasmus MC Cancer Institute, University Medical Center, 3008 AE Rotterdam, The Netherlands; 3 Department of Radiation Science and Technology, Delft University of Technology, Mekelweg 15, 2629 JB Delft, The Netherlands; 4 Department of Physics and Atmospheric Sciences, Dalhousie University, Sir James Dunn Bldg., Halifax, NS B3H 4J5, Canada

**Keywords:** hybrid nanoparticles, iron oxide, palladium, thermal decomposition, magnetization, superparamagnetism, hyperthermia, heating power

## Abstract

Heterostructured magnetic nanoparticles show great potential for numerous applications in biomedicine due to their ability to express multiple functionalities in a single structure. Magnetic properties are generally determined by the morphological characteristics of nanoparticles, such as the size/shape, and composition of the nanocrystals. These in turn are highly dependent on the synthetic conditions applied. Additionally, incorporation of a non-magnetic heterometal influences the final magnetic behavior. Therefore, construction of multifunctional hybrid nanoparticles with preserved magnetic properties represents a certain nanotechnological challenge. Here, we focus on palladium/iron oxide nanoparticles designed for combined brachytherapy, the internal form of radiotherapy, and MRI-guided hyperthermia of tumors. The choice of palladium forming the nanoparticle core is envisioned for the eventual radiolabeling with ^103^Pd to enable the combination of hyperthermia with brachytherapy, the latter being beyond the scope of the present study. At this stage, we investigated the synthetic mechanisms and their effects on the final magnetic properties of the hybrid nanoparticles. Thermal decomposition was applied for the synthesis of Pd/Fe-oxide nanoparticles via both, one-pot and seed-mediated processes. The latter method was found to provide better control over morphology of the nanoparticles and was therefore examined closely by varying reaction conditions. This resulted in several batches of Pd/Fe-oxide nanoparticles, whose magnetic properties were evaluated, revealing the most relevant synthetic parameters leading to promising performance in hyperthermia and MRI.

## 1. Introduction

Nanomaterials have long been of high scientific interest due to their unique properties arising from the versatile nanoscale structural characteristics, and thus, functional performance. In recent years, research has increasingly focused on the development of complex heterostructured nanosystems that combine two or more functional species within a single entity. Such hybrid nanoparticles (NPs) have an immense potential for a wide range of applications thanks to the superior inherent properties and the accompanying multifunctionality. 

Iron oxide nanoparticles (Fe-oxide NPs) are of particular interest as they exhibit tunable magnetic properties suitable for various biomedical applications [1]. In their superparamagnetic regime, Fe-oxide NPs are able to accelerate the transverse relaxation time (*T*_2_) of bulk water protons in the presence of an external magnetic field and can therefore be used as contrast agents in magnetic resonance imaging (MRI) [2]. At the same time, they can also generate heat when exposed to an alternating magnetic field (AMF) allowing hyperthermia or thermal ablation of the surrounding tissues [3]. Effectiveness of these performances is highly dependent on the chemical (Fe_2_O_3_/Fe_3_O_4_) and morphological (size/shape) characteristics of the Fe-oxide NPs. Additional functionalities, such as drug delivery [4], optical imaging [5], and radionuclide imaging [6], are typically realized through appropriate functionalization of the surface. On the other hand, insertion of a heterometal directly into the NPs endows the hybrid materials with interesting features, such as antimicrobial activity (silver/Fe-oxide) and imaging by computed tomography (gold/Fe-oxide) [7], or chelate-free radiolabeling (zirconium-89/Fe-oxide) [8]. Since these modifications involve interference with Fe-oxide lattice, the preservation of magnetic properties becomes a challenge, and extreme caution is needed to precisely control the synthesis of such hybrid nanomaterials [9].

Among the strategies to produce inorganic metal-based NPs, thermal decomposition (TD) is considered one of the most appropriate methods to generate highly crystalline, monodispersed NPs with good morphology control compared to other traditional wet-chemistry synthesis methods, such as co-precipitation [10], sol-gel [11] or microemulsion [12]. The TD process consists of thermolysis, in which a metal complex undergoes chemical decomposition in organic solvent with a high-boiling point in the presence of surfactants that stabilize the generated NPs [13]. A straightforward way to synthesize hybrid metal nanocrystals based on TD is the one-pot approach, where homogeneous nucleation and seed-assisted growth occur simultaneously [14]. On the other hand, the two-step seed-mediated method is considered a better alternative that allows for precise control of size and morphology, relying on the heterogeneous nucleation and growth of a second solid phase on the surface of the preformed so-called “seeds” introduced into the reaction mixture [15,16]. Additionally, the TD synthesis has a multitude of parameters, which enable great freedom in the design of NPs by tuning their size, morphology, and composition [13,17]. However, these parameters are co-dependent and, despite the great effort put into the research of synthesizing hybrid nanoparticles via TD, the mechanism of formation and the role of the diverse synthetic parameters to achieve different morphologies are still not fully understood [18]. Synthesis of hybrid Fe-oxide NPs containing a non-magnetic heterometal via TD has been extensively described for gold/Fe-oxide [16,18,19], but there are only a few reports on Pd/Fe-oxide systems, focusing on their application for catalysis, rather than on magnetic properties or evaluation of synthetic mechanisms [20,21,22,23]. 

In our work, we aim at exploiting the theranostic potential of Fe-oxide NPs, which simultaneously enable diagnosis (MRI) and treatment of tumors by means of combined hyperthermia and radiotherapy after injection of the NPs directly into the tumor mass as a brachytherapy procedure. The latter component can be realized by creating a palladium core in the magnetic Fe-oxide NPs containing its radioisotope (^103^Pd), which decays with a half-life of 17 days by electron capture/low energy X-rays (21 KeV), ideal for depositing therapeutic radiation in tumors [24,25,26]. Methods to incorporate ^103^Pd into Pd/Fe-oxide NPs have recently been investigated [27] and are out of scope at this point. Here, we explore the synthetic conditions for manufacturing Pd/Fe-oxide NPs via TD of iron pentacarbonyl and elucidate the effects of different reaction parameters on the morphological and physical properties of the NPs. Within this frame of reference, both one-pot and seed-mediated methods were investigated for the controlled production of Pd/Fe-oxide hybrid nanocrystals. Next, the seed-mediated method was studied closely by the systematic variation of the following parameters: the Fe-precursor/Pd-seed ratio, functionalization of the Pd-seeds, surfactant/Fe-ratio, heating rate and solvent. Lastly, the magnetic properties of the obtained hybrid NPs were investigated and the resulting characteristics plus the corresponding parameters were brought in relation with the best performance in magnetic hyperthermia and MRI. 

## 2. Materials and Methods

### 2.1. Reagents

Pd(II)-acetylacetonate (Pd(acac)_2_), 1,2-hexadecanediol, 1-octadecene (ODE), oleic acid (OA), oleylamine (OAm), iron pentacarbonyl (Fe(CO)_5_), ethanol, hexane, chloroform, Palladium (II) acetate, tert-butylamine-borane complex, n-dodecyl sulfide, toluene, diphenyl ether (DBE), dibenzyl ether (DBE), 1,2-distearoyl-sn-glycero-3-phosphoethanolamine-*N*-[carboxy(polyethylene glycol)-2000] sodium salt, powder (DSPE-PEG_2000_-COOH). All chemicals were used as received without further purification. All synthetic methods were conducted in a standard airless procedure.

### 2.2. Preparation of Pd/Fe-Oxide NPs via a One-Pot Synthesis Method

#### 2.2.1. Flower-like Pd/Fe-Oxide NPs

The NPs were synthesized via a one-pot synthesis method following a previously reported protocol [28]. Briefly, Pd(acac)_2_ (152 mg, 0.5 mmol), 1,2-hexadecanediol (646 mg, 2.5 mmol) and 20 mL octadecene were added to a 3-neck round-bottom flask and purged with nitrogen for 30 min under vigorous stirring. Subsequently, the reaction mixture was heated to 85 °C in approx. 2 min, after which OA (190 µL, 0.5 mmol), OAm (175 µL, 0.5 mmol) and iron pentacarbonyl (263 µL, 2 mmol) were injected via a septum in this exact order. The temperature of the reaction mixture under nitrogen atmosphere was brought instantly to 300 °C and maintained at this temperature for 30 min. After 30 min, the reaction system was left to cool down to room temperature and the Pd/Fe-oxide NPs were collected by addition of 30 mL ethanol and centrifugation for 6 min at 10,500 RPM (11,830× *g*). The procedure was repeated several times with ethanol and one or two times with a combination of ethanol and hexane in equal volumes. Lastly, the Pd/Fe-oxide NPs were dried by a gentle flow of compressed air and stored as such until further use. The final product was redispersible in organic solvents such as toluene, hexane, chloroform. 

#### 2.2.2. Popcorn-like Pd/Fe-Oxide NPs

The NPs were produced also via the one-pot synthesis method following a previously reported protocol [28] and described above, but with slight modifications. Pd(acac)_2_ (152 mg, 0.5 mmol), 1,2-hexadecanediol (646 mg, 2.5 mmol) and 20 mL octadecene were added to a 3-neck round bottom flask and purged with nitrogen for 30 min under vigorous stirring. Subsequently, the reaction mixture was heated to 85 °C in approx. 2 min. At 85 °C, OA (637 µL, 2 mmol), OAm (658 µL, 2 mmol) and iron pentacarbonyl (263 µL, 2 mmol) were injected via a septum in this order. The reaction mixture was further heated to 300 °C with a 5 °C/min heating rate under a nitrogen blanket and maintained at this temperature for 30 min. After 30 min, the reaction system was left to cool down to room temperature and the Pd/Fe-oxide NPs were collected by addition of 30 mL ethanol and centrifugation for 6 min at 10,500 RPM (11,830× *g*). The procedure was repeated several times with ethanol and one or two times with a combination of ethanol and hexane in equal volumes. Lastly, the Pd/Fe-oxide NPs were dried by a gentle flow of compressed air and stored as such until further use. The final product was redispersible in organic solvents such as toluene, hexane, chloroform. 

### 2.3. Preparation of Pd-Seeds

#### 2.3.1. Preparation of Oleylamine Capped Pd NPs (Seeds)

OAm capped Pd-seeds were prepared based on previously published protocols [29,30]. Briefly, palladium (II) acetate (56 mg, 0.249 mmol) was added to 15 mL OAm in a 3-neck round-bottom flask. The reaction mixture was heated to 60 °C in 10 min under a stream of nitrogen gas and vigorous stirring. In parallel, t-butylamine-borane complex (130 mg, 1.495 mmol) was dissolved in 3 mL OAm and injected into the reaction mixture via a septum, once the temperature reached 60 °C. After addition, the reaction mixture was further heated to 90 °C with a heating rate of 3 °C/min and kept at this temperature for 60 min. After 60 min, the reaction system was left to cool down to room temperature and the Pd NPs were collected by addition of 30 mL ethanol and centrifugation for 8 min at 10,500 RPM (11,830× *g*). The Pd NPs were stored as such until further use. The final product was redispersible in organic solvents such as toluene, hexane, chloroform. 

#### 2.3.2. Preparation of *N*-dodecyl Sulfide Capped Pd NPs (Seeds)

*N*-dodecyl sulfide capped Pd NPs were synthesized following a previously published protocol [31]. Palladium (II) acetate (100 mg, 0.89 mmol) together with *n*-dodecyl sulfide (0.825 g, 2.23 mmol) were added to a 3-neck round-bottom flask containing 25 mL toluene. The solution was heated to 90 °C under a nitrogen blanket and kept at this temperature for 3 h. After 3 h, the reaction system was left to cool down to room temperature and the solvent was removed under vacuum. The resulting precipitate was washed twice with acetone by centrifugation for 5 min at 10,500 RPM (11,830× *g*). The Pd-seeds were left to dry and kept in a scintillation glass vial until further use. The final product was redispersible in organic solvents such as toluene, hexane, chloroform.

### 2.4. Preparation of Pd/Fe-Oxide NPs via a Seed-Mediated Method

For the synthesis of Pd/Fe-oxide NPs, an iron oxide coat was added via a classical thermal decomposition procedure on top of the pre-made Pd-seeds, by following a previously published protocol with slight modifications [32]. OAm capped Pd NPs (20 mg, 0.188 mmol) were dispersed in approximatively 0.5 mL hexane and sonicated for 5 min. Next, the Pd-seeds in hexane were added to a 3-neck round bottom flask containing 20 mL octadecene, OA (1 mL, 3.14 mmol) and OAm (1 mL, 3.14 mmol). The reaction mixture was slowly heated to 120 °C under nitrogen flow and vigorous stirring and left at this temperature for 30 min to ensure the complete removal of hexane. Subsequently, iron pentacarbonyl (150 µL, 1.141 mmol) was injected into the reaction vessel via a septum and the reaction system was heated to 300 °C with a heating rate of 5 °C/min and kept at this temperature for 30 min. After 30 min, the reaction system was left to cool down to room temperature and the Pd/Fe-oxide NPs were collected by addition of 30 mL ethanol and centrifugation for 6 min at 10,500 RPM (11,830× *g*). The procedure was repeated several times with ethanol and one or two times with a combination of ethanol and hexane in equal volumes. Lastly, the Pd/Fe-oxide NPs were dried by a gentle flow of compressed air and stored as such until further use. The final product could be redispersed in organic solvents such as toluene, hexane, chloroform.

### 2.5. Dispersion of Pd/Fe-Oxide NPs in Aqueous Media with DSPE-PEG_2000_-COOH

The previously synthesized Pd/Fe-oxide NPs were transferred into water by modification with DSPE-PEG_2000_-COOH following a slightly modified protocol presented elsewhere [33]. Briefly, 1 mg Pd/Fe-oxide NPs were dispersed via ultrasonication in 1 mL chloroform containing 1.5 mg of DSPE-PEG_2000_-COOH. The vial containing the mixture was then left open for 24–48 h for the slow evaporation of the solvent, until a pasty precipitate remained on the bottom of the vial. The residual solid was heated to 80 °C for 10 min in a vacuum oven, to ensure complete removal of chloroform. Next, 1 mL of Milli-Q water was added to the precipitate and sonicated for 15 min until a colloidal suspension in aqueous media was obtained. The colloidal suspension of NPs was pipetted to Eppendorf vials and the unbound polymer and excess lipids were purified by centrifugation for 1 h at 19,600 RPM (30,000× *g*) and subsequent removal of the supernatant. This procedure was repeated twice. Lastly, the NPs were collected in 1–2 mL of Milli-Q water in an Eppendorf and kept as such for further use. 

### 2.6. Characterization

Particle size, size distribution and morphology of the samples were determined by transmission electron microscopy (TEM), using a 120 kV Jeol_JEM1400 microscope. All samples for TEM were prepared by drop-casting a diluted nanoparticle suspension in organic solvents such as hexane on a Quantifoil R1.2/1.3 Cu300 grid and evaporating the solvent at room temperature. The mean diameter and the size distribution of the samples were obtained by statistical analysis over 500–1000 NPs, by analyzing the obtained TEM images with ImageJ software. The elemental mapping analysis was done with an Oxford Instruments EDX detector X-MAX^N^ 100TLE on the same grids used for TEM.

Magnetic characterization by superconducting quantum interference device (SQUID), was carried out on an MPMS XL magnetometer from Quantum Design, using about 1–2 mg of dried NPs powder. The hysteresis loops M(H) obtained under continuously varying applied magnetic field up to a maximum of ±60 kOe at 5 K and 300 K were used for evaluation of saturation magnetization (M_s_) and coercivity (H_c_). Zero-field-cooling (ZFC) and field-cooling (FC) magnetization curves were measured at 1 kOe in the temperature range 5–370 K. The sample was cooled from 370 K down to 5 K in the absence of magnetic field. Once the lowest temperature was reached, magnetic field was set to 1 kOe and magnetization was measured as the temperature was increased to obtain the ZFC curve. When back at high temperatures, the sample was cooled down again while a magnetic field was applied. The magnetic NPs were progressively blocked along the magnetic field axis, and the FC magnetization was measured. The blocking temperature (T_B_) was determined as the maximum on the ZFC curve. 

Transmission ^57^Fe Mössbauer spectra were collected at 300 K with a conventional constant-acceleration spectrometer using a ^57^Co (Rh) source. Velocity calibration was done using an α-Fe foil. The spectra were fitted using the Mosswinn 4.0 program [34].

The heating power measurements were performed using the Magnetherm Digital, manufactured by Nanotherics, using a 50 mm coil device. All SLP values were measured using an alternating magnetic field with a frequency of 346 kHz and a field strength of 23 mT. Two glass-fiber optic thermometers were used for temperature measurement [Osensa PRB-G40_2.0M-STM-MRI] to measure both the core and bottom temperature of the sample. The core temperature measured was used for SLP calculations. For determination of SLP, a sample of 1 mL of NPs in suspension was inserted in an isolated sample holder, to reduce heat loss to the environment, and placed in the middle of the coil. The temperature was equilibrated until it varied less than 0.05 °C/min before the measurement was conducted. The sample was then exposed to the magnetic field. 

Transversal relaxation times (*R*_2_) of water protons in the presence of magnetic NPs were measured on a Bruker Avance-500 NMR using Carr-Purcell-Meiboom-Gill (CPMG) pulse sequence with variable spin-echo train and echo time of 0.5 ms. 

The SLP-values and *r*_2_-relaxivities of the samples were calculated using the concentrations of iron obtained from the ICP-OES data performed on the same samples after destruction of NPs.

## 3. Results and Discussion

### 3.1. Nanoparticles Synthesis

**Scheme 1:**
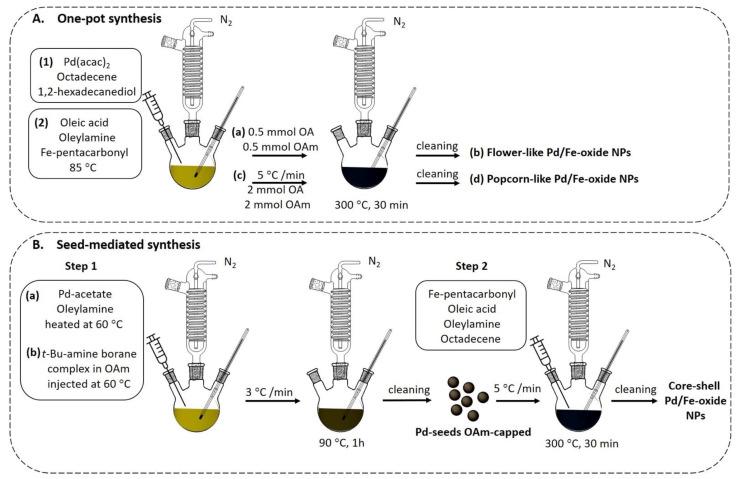
Synthetic routes for the preparation of hybrid Pd/Fe-oxide NPs via (**A**) one-pot procedure and (**B**) seed-mediated synthesis under conditions further notified as Exp_standard.

The preparation of Pd/Fe-oxide NPs was investigated by following two routes: one-pot procedure [28] and seed-mediated synthesis, and the resulting hybrid NPs were compared in terms of their size and morphology. Generally, the one-pot procedure (Scheme 1A) involves in situ formation of metal seeds by means of homogeneous nucleation as a result of thermal decomposition of a precursor, in this case Pd-acetylacetonate (Pd(acac)_2_). Subsequent injection of the second metal precursor, iron pentacarbonyl (Fe(CO)_5_), into the reaction mixture and further temperature increase, trigger the second thermal decomposition reaction occurring on the surface of the already formed Pd-seeds. As the result, the final morphology of the hybrid NPs is determined by the second nucleation process in which iron oxide is deposited on the surface of Pd-seeds.

**Figure 1:**
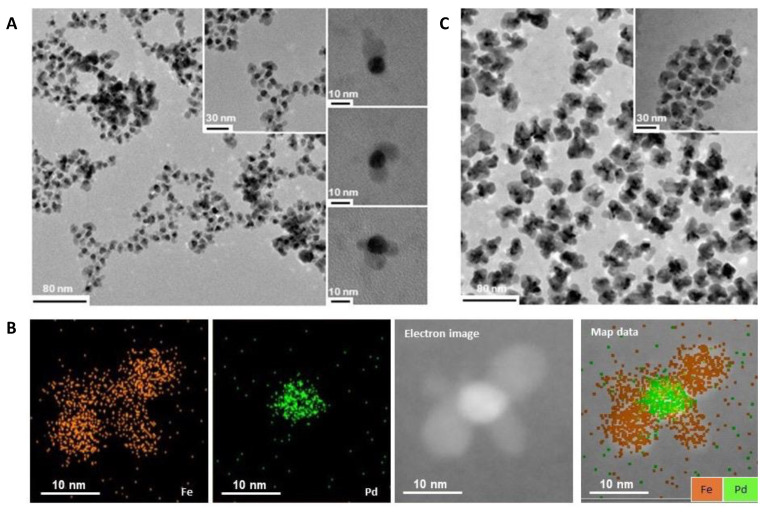
Characterization of Pd/Fe-oxide NPs obtained with one-pot synthesis resulting in: flower-like NPs—TEM images (**A**), incl. the three specific types of shapes they undertook (insets), and EDS elemental mapping with individual signals for Fe and Pd (**B**), and popcorn-like NPs—TEM images (**C**).

For instance, introducing 0.5 mmol OA and 0.5 mmol OAm during the reaction and an instant increase in the temperature to 300 °C (Scheme 1A(a,b)) results in flower-like Pd/Fe-oxide NPs (Figure 1A). As depicted in the right inset, the NPs exhibit 3 different morphologies as they consist of a 7 nm Pd-core with one, two or three Fe-oxide lobes of approximately 9 nm. In the bright-field TEM image (Figure 1A), the darker areas correspond to palladium, while lighter contrast represents Fe-oxide. This occurs due to the different electron penetration efficiency on the Pd compared to Fe-oxide. To confirm this, EDS elemental mapping was performed on the flower-like Pd/Fe-oxide NPs visualizing the individual signals for Pd and Fe (Figure 1B).

To better understand the effect of the synthetic conditions on the morphology of final hybrid Pd/Fe-oxide NPs, the one-pot synthesis was repeated with slight modifications, in which higher amounts of OA (2 mmol) and OAm (2 mmol) were used, and the 300 °C, necessary for the second nucleation reaction, were reached gradually, increasing the temperature by 5 °C/min (Scheme 1A(c,d)). As a result of this experiment, Pd/Fe-oxide NPs of about 30 nm in size were obtained in a popcorn-like morphology, with Fe-oxide lobes randomly arranged around the Pd-core, as shown in Figure 1C. Despite the same principle of NPs formation in both experiments, in the case of popcorn-like heterostructures the Pd-cores were found to be less uniform and completely coated with Fe-oxide (Figure 1C), which on its turn resulted in a decreased homogeneity within the sample. Clearly, the simultaneously occurring homogeneous nucleation and seed-assisted growth are two competing processes, which may be integrated under similar experimental conditions, but the individual optimal parameters can differ significantly and the control over the synthesis is challenging. This can lead not only to polydispersity in terms of size and shape, but also to diversity in the crystallinity and internal structure they present. Indeed, as demonstrated, both experiments based on the one-pot procedure resulted in Pd/Fe-oxide NPs with wide variations in size and shape.

**Figure 2:**
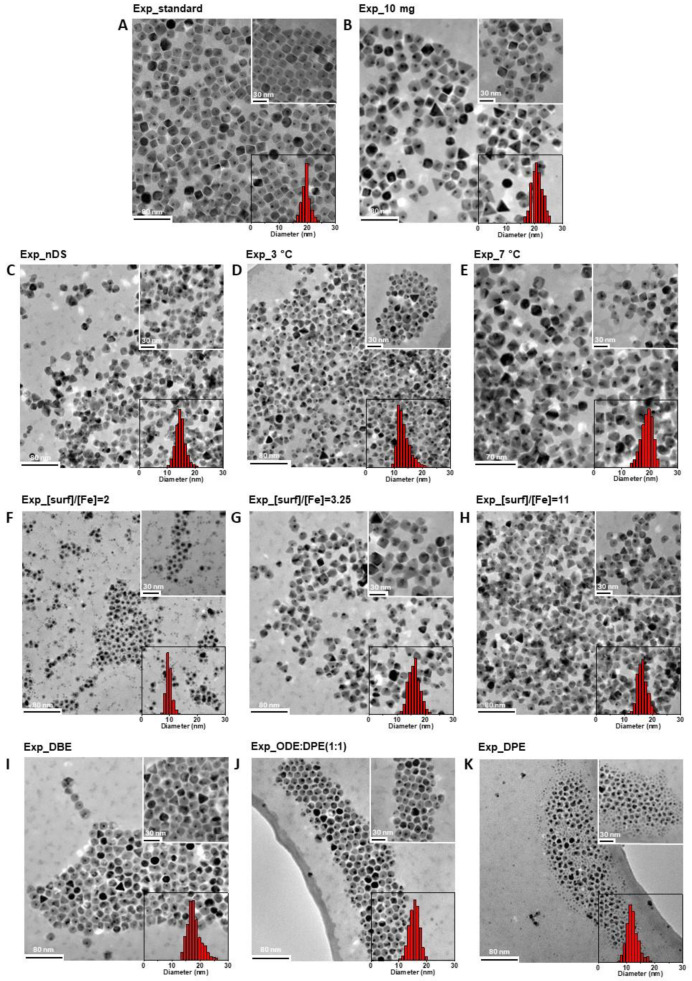
TEM images of Pd/Fe-oxide NPs obtained through seed-mediated synthesis, including expansions (insets **top right**) and the corresponding size-distributions (insets **bottom right**). Characteristics of NPs batches are summarized in Table 1.

The aforementioned uniformity issues could be resolved by separating the nucleation processes of the two metals. This can be achieved by the two-step seed-mediated growth process, which offers the possibility to prevent premature homogeneous nucleation of the second metal, by deposition of its atoms on the preformed seeds with well-defined characteristics [15]. For better understanding, the seeded-growth is based on a key principle of the classical nucleation theory, which states that the energy barrier that needs to be overcome for a certain material to heterogeneously nucleate onto a pre-existing seed is lower than the activation energy necessary to induce the corresponding homogeneous nucleation [17]. In simpler terms, if pre-made seeds are inserted into a reaction mixture during NPs synthesis, it is energetically more favorable for monomers to undergo phase transformation at the surface of a seed, than to self-nucleate. Therefore, a seed-mediated growth process was applied for the synthesis of another batch of Pd/Fe-oxide NPs (Scheme 1B) in which the iron precursor (Fe(CO)_5_) was decomposed and iron atoms were deposited on the surface of the pre-made OAm-capped Pd-seeds with the average diameter of 5 nm (Figure S1A). As the result, the iron-oxide formed a coating on top of the Pd-seeds, leading to monodisperse Pd/Fe-oxide NPs with spherical-squared morphology and sizes around 19 nm, as can be seen in the TEM images presented in Figure 2A. 

Both, the one-pot synthesis method and the seed-assisted growth method resulted in heterogeneous nucleation of Fe-oxide on the Pd-seeds by TD using Fe(CO)_5_ in 1-octadecene with OA and OAm added as ligands. However, a lower monodispersity of the nanocrystals synthesized via the one-pot process can be observed when compared to the nanocrystals made via the seed-mediated growth process. This does not come as a surprise, and based on the previously stated principles of TD, the seed-mediated growth procedure was more easily controllable than the one-pot synthesis method, offering hybrid NPs with higher monodispersity. Therefore, the one-pot synthesis method was not further investigated, and all efforts were devoted to carefully study the synthetic process of the seed-mediated growth in order to better understand the mechanism and to achieve high control over the morphology of the obtained hybrid nanocrystals. Starting from the reaction conditions depicted in Scheme 1B (defined as Exp_standard) that generated the Pd/Fe-oxide NPs presented in Figure 2A, different synthetic conditions were investigated to determine the key parameters involved in the control of the nucleation and successive growth of Fe-oxide on top of the Pd-seeds. The overview of the conducted experiments along with the corresponding conditions are summarized in Table 1.

### 3.2. Tuning Parameters in the Seed-Mediated Growth Procedure

#### 3.2.1. Effect of the [Fe]/[Pd] Ratio

The effect of the [Fe]/[Pd] ratio was investigated by conducting an experiment under conditions summarized in Table 1 as Exp_10mg, using a smaller amount of pre-made OAm-capped Pd-seeds (10 mg) compared to that used in Exp_standard (20 mg). As the result, Pd/Fe-oxide NPs with spherical-squared morphology and sizes around 21 nm were formed (Figure 2B). The possibility to tune the size of NPs by varying the precursor/seed ratio, in our case [Fe]/[Pd], is known [15]. Indeed, in the case of Pd/Fe-oxide hybrid nanocrystals obtained using only half of the number of Pd-seeds compared to Exp_standard, the NPs presented a slight increase in their average size. This can be explained as introducing a lower number of Pd-seeds into the reaction translates into a smaller surface area available for Fe-oxide deposition forming a thicker Fe-oxide layer, resulting in overall larger sizes of Pd/Fe-oxide NPs. 

Notably, no major influence on the morphology of the hybrid nanocrystals was observed, meaning that both the thermodynamic and kinetic control of the reaction were ensured in order for the original shape to be maintained [3]. As the overall surface area of the nanocrystals increases with size, more capping agent will be gradually depleted from the solution, therefore, excess of surfactant is often necessary. This was ensured through the experiment, even though the [surf]/[Fe] ratio was not specifically adjusted in this case.

As discussed earlier, seed-mediated growth offers an effective way to avoid homogeneous nucleation by deposition of the atoms on preformed seeds because of the lower energy barrier. However, when the monomer concentration exceeds a certain threshold, both homogeneous and heterogeneous nucleation can occur concomitantly, leading to nanocrystals with diverse morphologies, sizes, shapes and internal structures [3]. In the specific case of Exp_10mg, next to the Pd/Fe-oxide NPs, a second population of small Fe-oxide nanocrystals with the diameter of around 9 nm could be found (Figure S2). 

#### 3.2.2. Effect Pd-Seeds Coating Layer

Surfactants play a crucial role in the formation of the intermediate complexes during the synthesis of nanocrystals by TD. These ligands protect the surface and confine the size of the obtained nanostructures to nanoscale by forming a dynamic layer, which not only allows to control the growth of the primary nanocrystals, but also the nucleation of another inorganic phase onto their surface, leading to hybrid NPs [18]. Therefore, requirements for the surface ligand include compatibility with growth-conditions and the nature of its binding to the surface of the primary seeds, which should generally not be too strong [35]. In order to investigate how different surfactant layers of the Pd-seeds used during the seed-mediated growth synthesis influence the final morphology of Pd/Fe-oxide NPs, Exp_standard was repeated using Pd-seeds with n-dodecyl sulfide (nDS) instead of OAm as a capping agent under synthetic conditions summarized in Table 1 as Exp_nDS. These nDS-coated Pd-seeds have spherical shapes, but smaller sizes (3 nm) (Figure S1B) compared to OAm-coated Pd-seeds (5 nm) used for Exp_standard (Figure S1A).

As presented in Figure 2C, Pd/Fe-oxide NPs with an average size of 15 nm were obtained employing nDS coated Pd-seeds, compared to the 20 nm Pd/Fe-oxide NPs obtained using OAm coating of Pd-seeds in Exp_standard. Besides the decrease in size, the final NPs also exhibited a large diversity in morphology. The nanocrystals undertook spherical, squared, triangled, and flower-like morphologies, which ultimately translates in higher size distribution. 

As all other reaction conditions were kept the same during the synthesis, it is obvious that the new ligand nDS present on the Pd-seeds either affected the growth-phase or played a role in limiting the access to Pd-seeds due to the strong binding to their surface. Similar effects of the sulfur-containing ligands hindering the epitaxial overgrowth of metal oxides on noble metal nanocrystals have been reported in literature [35]. In contrast, as a weak ligand, OAm is known to bind less strongly to the surface of noble metals, presumably facilitating the growth-phase more than the nDS ligand, which on its turn explains the smaller sizes of the NPs obtained with the latter surfactant. 

Ligands also play an important role in the shape-control of the NPs, modulating the surface energy of a specific facet and favoring growth in certain crystallographic planes. The strength of binding of each capping agent is different for various facets, and in some cases, an exclusive facet is preferred over another. Lastly, a facet that is selectively capped will result in its dominant expression during growth. This means that a certain morphology will be preferred over others [15]. One of the examples of capping ligands determining the morphology of NPs is the combination of carboxylic acid/amine ligands, known to yield cubic structures [35]. Since such great diversity in shape was noted for the Pd/Fe-oxide NPs obtained with nDS-capped Pd-seeds, this ligand most likely has neither an exclusive facet for binding, nor a clear effect on specific morphology, as OAm does. 

#### 3.2.3. Effect Heating Rate

As nanoparticle synthesis based on TD requires high temperatures to trigger the decomposition of the iron precursor, the rate of temperature increase is another important parameter among the wide range of variables that can influence this process. Cotin et al. considered that the heating rate used to reach the growth-temperature is probably even more important than the boiling temperature of the solvent in which the synthesis is conducted [13]. During this study, the influence of the heating rate was investigated by running experiments with two variable rates implemented to reach 300 °C, one lower and one higher than 5 °C/min applied in Exp_standard. The experimental details for these batches of Pd/Fe-oxide NPs are summarized in Table 1 as Exp_3 °C and Exp_7 °C for experiments with the heating rates of 3 °C/min and 7 °C/min, respectively. 

The Pd/Fe-oxide NPs resulted from the experiment with a heating rate of 3 °C/min exhibited an average size of 13 nm (Figure 2D), which is smaller than the average size of the Pd/Fe-oxide nanoparticles obtained through the experiments in which higher heating rates were employed. A change in morphology was also noted in this case, as most of the NPs undertook a dumbbell-like shape, and irregular shapes were more frequently encountered than in the other samples synthesized with higher heating rates. This also explains the broader size distribution found in Exp_3 °C. All these results are in agreement with those from other studies by Lassenberger et al. [36] and Cotin et al. [13], who also noted smallest core sizes and broadest size distribution for the NPs synthesized at low heating rates. Similar to these observations, our results suggest that the probable reason for this inhomogeneity comes either from multiple nucleation steps or as a consequence of the nucleation step not being quick enough due to the lower heating rate. Additionally, Ostwald ripening may also be the reason for the presence of both small and large NPs within the same sample. In this case, the smallest nanocrystals with the higher surface energy start to dissolve and the created monomers diffuse to the surface of larger NPs and participate in their growth further on. 

On the other hand, the nanocrystals produced via Exp_7 °C were found to be very similar in average size, morphology, and size distribution (Figure 2E) to the Pd/Fe-oxide NPs obtained from Exp_standard. The average size of the Pd/Fe-oxide NPs from Exp_7 °C was 19 nm, retaining a round-squared morphology. However, the fast heating rate causes production of many monomers, surpassing the minimum number necessary for nucleation and possibly overcoming the threshold for homogeneous nucleation [13]. As the result, this high burst of monomers compared to slower heating rates causes the undesired homogeneous nucleation in the form of Fe-oxide NPs within the sample (Figure S3).

#### 3.2.4. Effect Surfactant to Iron Ratio

It is known that the surfactant/metal ratio ([surf]/[Fe]) has an influence on the size of the final NPs, and specifically, that a decrease in the amount of surfactant for the same iron precursor concentration is expected to produce smaller nanoparticles [29]. To investigate this, experiments were conducted under standard conditions, but varying the amount of surfactant introduced in the reaction mixture. Two experiments with a lower and one experiment with a higher [surf]/[Fe] ratio than that used for Exp_standard were performed. The synthetic conditions are summarized in Table 1 as Exp_[surf]/[Fe] = 2, Exp_[surf]/[Fe] = 3.25 and Exp_[surf]/[Fe] = 11. 

The experiment in which a [surf]/[Fe] ratio of 2 was employed resulted in Pd/Fe-oxide NPs with 10 nm average size and a fraction of Fe-oxide nanocrystals around 4 nm (Figure 2F). Increasing the [surf]/[Fe] ratio to 3.25, resulted in an increase in the average size of Pd/Fe-oxide NPs to 16 nm, but no homogeneous nucleation was noticed in this case (Figure 2G). As expected, both experiments with [surf]/[Fe] ratios being smaller compared to those in Exp_standard (5.5) yielded Pd/Fe-oxide NPs with average sizes smaller than 20 nm. Interestingly, doubling the [surf]/[Fe] ratio from 5.5 in Exp_standard to 11 (Exp_[surf]/[Fe] = 11), led to Pd/Fe-oxide NPs with a lower average size of 16 nm (Figure 2H). Possibly, a larger amount of ligand used in Exp_[surf]/[Fe] = 11 increased the stability of the iron precursor, and consequently the decomposition temperature. Additionally, increased surfactant concentration may also mean that there are simply more ligand molecules at the surface and around the formed nuclei, which also contributes to either slowing down or inhibiting the growth-process. Consequently, this increase in amount of surfactant influences the growth-time, which also translates into a lower concentration of monomers available for the growth-step compared to Exp_standard. 

Notably, the Pd/Fe-oxide NPs obtained from Exp_[surf]/[Fe] = 11 had an average size very close to those obtained from Exp_[surf]/[Fe] = 3.25. However, the experiment with a [surf]/[Fe] ratio of 11 offered a better size distribution, while NPs prepared using a [surf]/[Fe] ratio of 3.25 exhibited high polydispersity. These observations are in agreement with Lassenberger et al. [36], who observed that the temperature for onset of rapid growth decreases with decreased surfactant concentration. Moreover, an increase in the polydispersity during the growth-phase was observed when using smaller amounts of surfactant compared to the cases when NPs were synthesized with higher [surf]/[Fe] ratios [29]. Based on the results obtained from this series of experiments, it can be hypothesized that the mean size of the NPs does increase with the amount of ligand up to a critical value, after which the linear behavior fails (Figure S4). The mechanism underlying this phenomenon is however still not clearly formulated and conflicting results obtained through numerous studies on the stoichiometric ratio ligand/precursor with similar reaction mixtures [13] make it difficult to compare our results with those from literature. 

The chemical nature of the ligand has also a direct effect on the reaction mechanism [13], and in order to study this, an experiment was conducted in the presence of OAm only with [OAm]/[Fe] = 5.5, as in Exp_standard. The experiment resulted in a product composed of large aggregates, from which the majority is Fe-oxide NPs (Figure S5A). In parallel, an experiment in the presence of OA only with [surf]/[Fe] = 5.5, as in Exp_standard was conducted in order to study the influence of OA only on the NPs outcome. The experiment resulted in no core–shell Pd/Fe-oxide NPs either (Figure S5B). Amines are known to ease the decomplexation of the oleates coordinated to the iron, while the influence of carboxylic acid ligands can be quite complex as they can stabilize the precursors and have an effect of the monomer formation [13,37]. The results obtained in experiment Exp_OAm and Exp_OA suggest that using OAm or, respectively, OA as the only ligand is not sufficient for obtaining well-formed Pd/Fe-oxide NPs. Therefore, we can conclude that both OAm and OA are necessary for the formation of well-defined Pd/Fe-oxide hybrid NPs, with both surfactants playing a role against aggregation and in the control of the iron oxide domains.

#### 3.2.5. Effect Solvent/Temperature

The iron precursor decomposes at a wide range of temperatures, meaning, the higher the temperature during synthesis, the more monomers are formed, which results in bigger NPs. Therefore, the NPs size depends on and increases with the boiling point of the solvent, as demonstrated in literature [37,38,39,40]. In order to verify this phenomenon for our hybrid NPs, the growth-mediated synthesis was conducted under standard conditions, but using solvents with boiling temperatures lower than that of ODE (315 °C) used in Exp_standard. Three batches of NPs were prepared in DBE (b.p. 298 °C), a mixture of ODE and DPE (1:1, b.p. ± 270 °C), and DPE (b.p. 258 °C). The corresponding TEM images and size distributions are depicted in Figure 2 (I, J and K, respectively). As expected, the mean diameter of the Pd/Fe-oxide NPs decreased with the decrease in the boiling point of the solvent (Table 2), confirming that the growth rate essentially depends on the temperature in which the reaction is conducted. 

However, the final size of the NPs is also dependent on the nature of the solvent. Observations by Cotin et al. reveal that the solvent is able to interact with the metal precursor and affect the monomer formation rate depending on its chemical properties [13]. Thereby, a polar solvent is more likely to destabilize the formed complex and yield smaller NPs, while the non-polar solvents stabilize the complex and condition larger NPs. Baaziz et al. drew the same conclusion about the nature of the solvent governing the nucleation temperatures, and therefore, it is fair to assume that the solvent plays a key role in the stability of the complex and its decomposition kinetics [37]. Our results confirm these observations, as the largest NPs were formed in Exp_standard, with the least polar solvent, ODE. Oppositely, DPE, the most polar solvent out of the four used through this study, led to the smallest Pd-Fe/oxide NPs (Figure 2K).

Another aspect presented in literature is that the polarity of the solvent can influence the morphology of the NPs. It is suggested that multinucleation may be favored by the use of slightly polar solvents, such as diphenyl ether or benzyl ether. However, in non-polar solvents, such as ODE, the Fe-oxide domain drains electrons from the seeds on which it grows and disables them from potential polynucleation, resulting in a single Fe-oxide domain [16,19,41]. Contrastingly, the results of the experiments performed in different solvents presented by Wei et al. suggest that neither the polarity of the solvent, nor the saturation of the solvent molecules play a role in the final morphologies of the synthesized hybrid NPs [19]. The reason behind this phenomenon is the crystallinity of the seeds on which the Fe-oxide was grown. 

However, most of the Pd/Fe-oxide NPs obtained from Exp_DPE do indeed present multiple iron domains, undertaking a flower-like shape (Figure 2K), as expected from a solvent with higher polarity [41]. Homogeneous Fe-oxide, found within Exp_DPE, is most likely formed due to the lower b.p. of DPE and, hence, the lower reaction temperature. These iron oxide species seem to have an amorphous character. However, complete crystallization of the Fe-oxide using DPE as a solvent seems to be possible with reaction time increased from 45 min to 3 h [42]. For comparison, we conducted Exp_ODE:DPE(1:1), in which a mixture of ODE and DPE in 1:1 ratio was used as solvent. Due to addition of non-polar ODE, the solvent mixture had a decreased polarity compared to DPE only, and the polynucleation was not favored anymore. Consequently, core–shell Pd/Fe-oxide NPs were obtained instead of flower-like (Figure 2J). 

Interestingly, core–shell NPs obtained from both, Exp_ODE:DPE(1:1) and Exp_DBE, were similar to those of Exp_standard. In the case of the former experiment, the Pd/Fe-oxide tended to undertake hexagonal shapes, while the latter one resulted in various shapes like spheres, triangles and even isolated flowers. This difference in shape-control can again be explained by the higher polarity of the ODE:DPE solvent mixture, compared to ODE. Based on the results obtained through this study, it is clear that the solvent has a significant effect on the outcome of the reaction by influencing the physical properties of the reaction surrounding media (e.g., polarity) and the stabilities of different reactants and intermediate species. This is generally in agreement with the studies conducted by others as mentioned earlier.

### 3.3. Magnetic Properties of Pd/Fe-Oxide NPs as a Function of Their Morphology 

**Figure 3:**
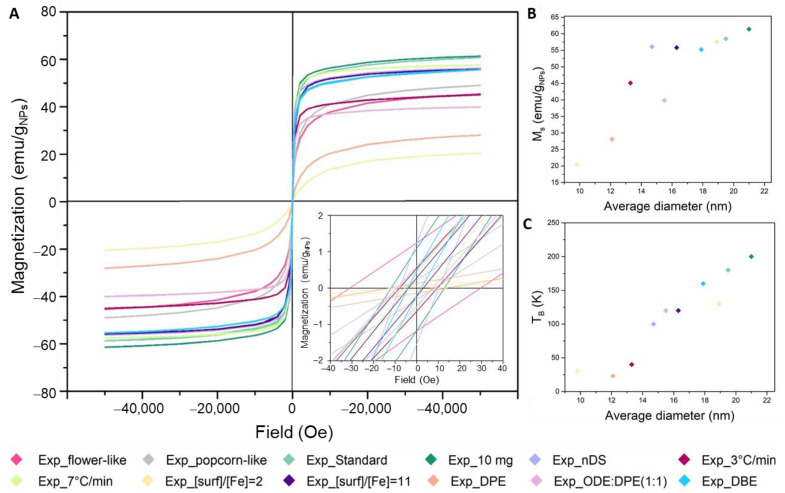
SQUID magnetometry of dry Pd/Fe-oxide NPs batches prepared under conditions summarized in Table 1: (**A**) M(H)_T_ curves measured at T = 300 K; the inset represents 2400× magnification of the *x*-axis showing coercivities (H_c_); (**B**) derived M_S_ at 300 K as a function of the average diameter; (**C**) T_B_ as a function of the average diameter of NPs.

Defining the optimal synthetic conditions leading to hybrid Pd/Fe-oxide nanocrystals with characteristics suitable for hyperthermia and MR imaging involves understanding the associated magnetic properties, and especially, their correlation with geometric data. Not less important is the possibility of the NPs to interact with each other if they are within the distances of a few Ångstroms, which can impact the collective magnetic response. Therefore, the static magnetic behavior of the prepared NPs was investigated by SQUID magnetometry. The measurements were conducted on the batches of NPs coated with oleic acid/oleylamine and in powder form. Magnetization curves resulting from alignment of NPs in the presence of an increasing magnetic field (up to 60 kOe) were measured at 300 and 5K, as presented in Figure 3A and Figure S6, respectively. The saturation magnetization (M_S_) could be determined by extrapolating the highest magnetization values to the ordinate axis, where the field approaches zero (Table 3). These values were found to be lower compared to the bulk magnetite (92 emu/g) [43] but corresponding to those of the NPs with the sizes around 20 nm [44], and close to the value of 84.5 emu/g measured from commercial magnetite fine powder [39]. The presence of Pd-core and organic layer at the surface were not accounted for, and therefore, it should be noted that the real M_S_ values expressed in emu/g of NPs should actually be higher.

The magnetization curves measured at 5K display a hysteretic behavior with substantial coercivities and remanence (Figure S6 and Table 3). This indicates typical behavior of superparamagnetic NPs below their blocking temperature (T_B_), above which the thermal fluctuations randomize magnetic moments leading to decreased M_S_ values as well as coercivities (Figure 3A-inset). As the result, at 300K the NPs exhibit characteristics of the superparamagnetic state, while the presence of some remained coercivities (Table 3) can be attributed to the complex interactions, such as frustrated order or spin canting phenomena, described for the surface functionalized core–shell structures [45]. 

Furthermore, the saturation magnetization of Pd/Fe-oxide NPs increases with the size of the nanocrystals (Figure 3B), which agrees with the literature [46]. Notably, this behavior fails for certain Pd/Fe-oxide batches, which can be explained by the shape variations of these NPs. Therefore, the M_S_ values for the ‘exotically’ shaped Exp_flower-like and Exp_popcorn-like NPs were excluded from comparison, as it is hard to determine their relevant average size. Moreover, it is challenging to draw an exact correlation between M_S_ and shape for the NPs with dissimilar volumes of Fe-oxide content [46]. Similarly, T_B_ (vide infra) is generally found to be proportional to the NPs volume/size [46]. This is a trend that we generally observe for our Pd/Fe-oxide NPs (Figure 3C) but once again, with certain exceptions. Moreover, we also need to consider the effect of dipolar interactions on the determined T_B_, as the NPs cannot be assumed fully isolated.

**Figure 4:**
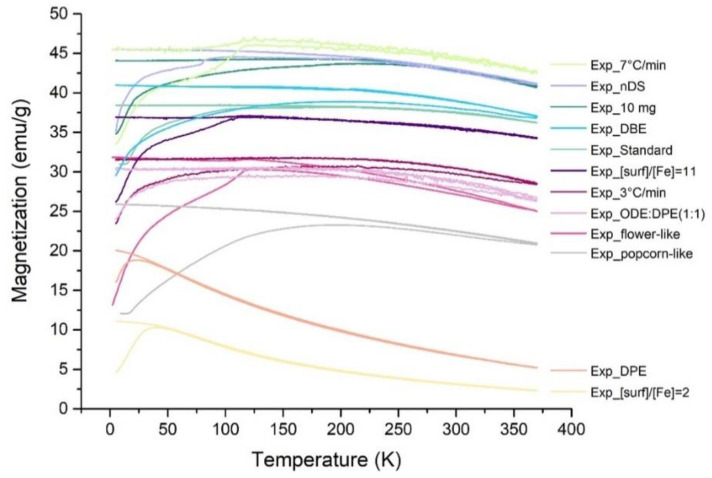
ZFC/FC-curves of the Pd/Fe-oxide NPs samples (abbreviations explained in Table 1) measured at 1 kOe.

More insights into the interparticle dipolar interactions could be obtained by measuring magnetization as a function of temperature under zero-field-cooling (ZFC) and field-cooling (FC) conditions (Figure 4). The NPs were cooled from 370 K to 5 K in the absence of magnetic field after which a small magnetic field (1 kOe) was applied. As the temperature increased (T < T_B_), the magnetic moments aligned along the direction of the field and the maximum magnetization was reached. This typical maximum in the ZFC-curve lays around the T_B_, which can then be calculated as the first derivative of zero magnetization [47] (Table 3). In some cases, such as for Exp_flower-like and Exp_popcorn-like NPs, the T_B_ is distributed over a certain temperature range, due to the size irregularities and interparticle interaction. The latter can also be concluded from the bifurcation of ZFC/FC curves above the T_B_ observed in several batches, except for Exp_standard, Exp_[surf]/[Fe] = 2, Exp_[surf]/[Fe] = 11, and Exp_DPE. At T > T_B_, the thermal energy exceeds that of the magnetic field, causing a random flip of magnetic moments, hindering a further increase in the measured magnetization. Thereby, the profiles of superparamagnetic NPs are characterized with a steep decrease in magnetization (e.g., Exp_DPE and Exp_[surf][Fe] = 2). As the transition magnetic NPs from single to multi-domain is expected at a critical diameter of 30–90 nm, depending on the shape, morphology, and magneto-crystalline anisotropy [48], we expect all batches of Pd/Fe-oxide, with Exp_popcorn-like as a possible exception, to be single domain. Indeed, all NPs present T_B_ way lower than room temperature, therefore, all Pd/Fe-oxide batches synthesized are in their superparamagnetic/single domain regime at room temperature. 

It is important to note that most studies presenting magnetic behavior of NPs are based on homogeneous Fe-oxide nanocrystals. Magnetic properties arise in the presence or absence of unpaired valence electrons and the distribution of cations in tetrahedral (A) and octahedral (B) sites within the inverse spinel crystal structures, such as magnetite (Fe_3_O_4_) and maghemite (Fe_2_O_3_) [46]. The presence of a second element, Pd in our case, influences the magnetic properties [49], and can be another reason why typical correlations fail for certain Pd/Fe-oxide NPs. Therefore, at this point it becomes interesting to take a closer look at the composition/crystallinity of the NPs, as magnetic properties can be influenced by (i) the ratio of low-magnetization maghemite to high-magnetization magnetite, and (ii) alteration of the chemical composition caused by OA bound strongly via its two carboxylic oxygens leading to magnetically inhomogeneous surface [50]. 

**Figure 5:**
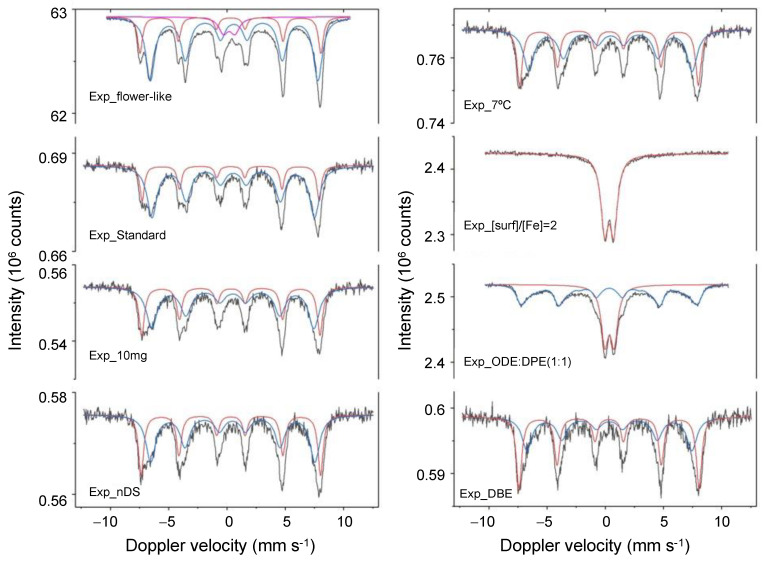
Mössbauer spectra of Pd/Fe-oxide NPs at 300 K.

The Mössbauer spectroscopy conducted on the NPs at 300K (Figure 5) reveals the hyperfine parameters determined by the position of Fe-ions within different lattice positions (Table 4). The first indication on the chemical composition at the A- and B-sites of magnetite can be derived from the isomeric shifts (IS). The charges of Fe^2+^ and Fe^3+^ ions at the octahedral B-site are delocalized and averaged to Fe^2.5+^ with IS of 0.67 mm s^−1^ expected for bulk magnetite. Therefore, the IS values measured for B-sites within the lattice that deviate downwards directly indicate the substitution of Fe^2+^ by Pd^2+^. 

With increasing Pd substitution levels (and decreasing B-site IS values)—the spectral contribution of the octahedral site is expected to decrease compared to the measured value in bulk magnetite (67%). This is indeed observed with samples Exp_10mg, Exp_nDS and Exp_7 °C, although the presence of small amounts of maghemite (overlapping with the A-site) cannot be excluded at this stage. In samples Exp_standard and Exp_flower-like the opposite effect is detected (in correlation with higher B-site IS values)—indicating substitution of Pd also at the A-site (preferentially). As the Mössbauer parameters do not directly correlate to the observed magnetic properties—it is conceivable that an optimum Pd substitution degree is achieved in the better performing Pd/Fe-oxide NPs. The presence of magnetite in sample Exp_nDS rather than maghemite detected for Exp_ODE:DPE(1:1) can explain the higher saturation magnetization of the smaller NPs (56.1 emu/g, 15 nm) compared to the bigger ones (39.9 emu/g, 16 nm). Additionally, the magnetite composition of the largest NPs (Exp_10mg (21 nm), Exp_standard (20 nm), and Exp_7 °C (19 nm) was confirmed based on the spectral contribution values (Table 4). 

Finally, the high crystallinity derived from the small Mössbauer line widths (Γ), the reasonably high M_S_ values saturating at relatively low fields, and the favorable hysteresis loops (especially of Exp_10mg, Exp_standard and Exp_7 °C NPs) suggest that the samples are of high magnetic quality, making them well suited for potential biomedical applications [51]. 

### 3.4. Magnetic Hyperthermia and Relaxivity

Hyperthermia entails increasing the temperature of an in vivo therapeutic system to a level that stimulates potential other therapies, such as chemo- or radiotherapy, in a temperature range of 41–46 °C, or that causes a targeted ablation and cell death, above 46 °C [52]. Magnetic particles can generate heat via different mechanisms in the surrounding regions when submitted to an alternating magnetic field. Depending on the size of the NPs, the heating can arise from hysteresis losses or, in the case of superparamagnetic nanoparticles, as are the Pd/Fe-oxide NPs presented in this study, the effects are attributed to Néel and Brownian relaxation mechanisms [46,53]. 

To evaluate the heating efficiency, we chose four of the Pd/Fe-oxide NPs batches introduced through this study: Exp_10mg, Exp_standard and Exp_7 °C with the highest values of saturation magnetization and, Exp_[surf]:[Fe] = 2 with the smallest sizes. Because the as-prepared hybrid nanocrystals were hydrophobic, the NPs were first transferred to aqueous media by providing their surface with hydrophilic DSPE-PEG_2000_-COOH. Next, samples containing approximatively 10 mg of dry Pd/Fe-oxide NPs dispersed in 1 mL of water were used while applying an AMF at the frequency of 346 kHz and a field strength of 23 mT. The efficiency of heating, expressed as specific loss power (SLP) [54], was calculated for each batch of Pd/Fe-oxide NPs based on the following equation:

(1) $$\mathrm{SLP}\;\left[W/g_{\mathrm{Fe}}\right]=\frac{C}{m_{\mathrm{Fe}}}\frac{\Delta T}{\Delta t}\;$$

where C is the heat capacity of the sample, m_Fe_ is the mass of iron in the colloidal solution determined by ICP-OES, and ΔT/Δt is the measured temperature increase with time. 

It has been suggested to use intrinsic loss power (ILP) to enable comparison of heating power measurements made at different magnetic field strengths/frequencies [55], which can be derived from SLP using Equation (2), in which SLP is in W/kg, *f* is the frequency of the alternating magnetic field in kHz and H is the magnetic field strength in kA/m.

(2) $$\mathrm{ILP}=\frac{\mathrm{SLP}}{f\cdot H^{2}}$$

The superparamagnetic Pd/Fe-oxide NPs from Exp_[surf]:[Fe] = 2 presented an SLP value of only 2 W/g_Fe_ (Table 3). This value is below the detection limit of the machine, meaning, no heat was generated by these NPs. This is in agreement with the study of Jeun et al., that established a threshold size (approx. 10 nm) below which the measured SLP is insufficient for hyperthermia applications [56]. Generally, the SLP values of magnetic Fe-oxide NPs are known to be proportional to the saturation magnetization of these NPs [57]. In our case, even with the inclusion of Pd as a second metal and the Fe-oxide as a coating, we noticed the same correlation of increasing SLP values with M_S_ as the Pd/Fe-oxide NPs with sizes of 19, 20 and 21 nm from Exp_7 °C, Exp_standard and Exp_10 mg, which produced SLP values of 102 W/g_Fe_, 113 W/g_Fe_, and 233 W/g_Fe_, respectively (Table 3). Of course, we are interested in the highest SLP values possible for efficient hyperthermia with a minimal dose of magnetic NPs introduced into the body, therefore, Exp_10mg had the most promising results. 

The SLP and ILP values are extensively used to characterize the heat generation ability of magnetic systems. As SLP is dependent on the frequency and strength of the magnetic field employed during the measurement, the ILP value is commonly used for comparison between magnetic nanoparticle heating powers measured at different laboratories [3,58]. However, it has been shown that ILP values of the same sample measured at several laboratories can differ up to 40% because of large systematic errors and lack of agreement in magnetic heating characterization [59]. This makes it hard to directly compare the results between our experiments and other different experimental setups. 

Once more, the presence of Pd in the composition of the NPs makes hard to compare our results with the results presented in literature on homogeneous Fe-oxide, and there are no other Pd/Fe-oxide NPs on which the heating efficiency has been investigated to the best of our knowledge. However, there are studies on Au/Fe-oxide NPs synthesized via thermal decomposition, such as the those reported by Efremova et al. [59] in which they present similar hybrid nanoparticles along with analogous method for transferring the hybrid nanoparticles in aqueous solution. Converting their maximum reported SLP value of Au/Fe-oxide NPs to the ILP results into 1.410 nHm^2^/kg, which is comparable to our results with a slightly higher ILP value (2.011 nHm^2^/kg) calculated for Exp_10mg. Still, this comparison is not fully relevant, as an AMF with different frequency and amplitude was applied during their heating studies, which still leads to differences in heating results [58].

MRI is a well-known diagnostic technique in medicine, and it is mostly applied to generate high-resolution anatomic images of organs and tissues. The principle is based on the nuclear magnetic resonance of the protons, whose relaxation can be altered by administration of contrast agents, which leads to contrast enhancement of the images. Superparamagnetic Fe-oxide NPs are superior candidates to decrease the *T*_2_-relaxation times of the protons in their proximity through generation of large magnetic field gradients resulting in a dark contrast in *T*_2_-weighted images [46,60]. As the Pd/Fe-oxide NPs presented promising magnetic properties, we performed *r*_2_ relaxivity (relaxation rate enhancement per mM of magnetic species) measurements. In accordance with the theory, only ultra-small Fe-oxide NPs (<10 nm) exhibit a *T*_1_-effect [61], which was not the case for the presented Pd/Fe-oxide NPs, thus the *r*_1_-measurements were omitted in this study. It has been shown that the contrast enhancement effects are directly related to the saturation magnetization value [46], thus the Pd/Fe-oxide NPs from Exp_[surf]:[Fe] = 2 were excluded, as their M_S_ was the lowest and the SLP value was insufficient for heating. Instead, we focused on the three Pd/Fe-oxide batches with the highest M_S_ values: Exp_10mg, Exp_standard and Exp_7 °C in aqueous suspensions and investigated their impact upon the *T*_2_ relaxation time. The resulting transverse relaxivities *r*_2_ measured for these three batches are summarized in Table 3. A typical increase in *r*_2_ values with increasing NPs size was observed [62] with the highest value of 440 mM^−1^ s^−1^ for Exp_10mg, making these NPs the most promising *T*_2_ contrast agents candidates, as the higher the relaxivity, the smaller the amount of NPs to be injected in the patient for high quality imaging. An inverse of the *T*_2_-relaxation time as a function of iron concentration for Exp_10mg can be found in Figure S7 in Supporting Information. 

## 4. Conclusions

We successfully synthesized Pd/Fe-oxide hybrid NPs with sizes between 9 and 30 nm via both, one-pot synthesis method and seed-mediated growth thermal decomposition procedures. As the hybrid nanocrystals obtained via the one-pot synthesis method presented less uniformity in terms of size and morphology, we focused on systematically investigating the seed-mediated growth procedure by varying one parameter at a time. These experiments offered important insights on the synthetic mechanism and on the control over reaction enabling various sizes and shapes of hybrid Pd/Fe-oxide NPs. 

Further, the magnetic properties of the multiple batches of Pd/Fe-oxide NPs were investigated. We noticed that the magnetic behavior of NPs is strongly size-dependent, and size is indeed one of the parameters that can be manipulated to tune the magnetic properties like saturation magnetization, coercivity, blocking temperature, etc. However, most of the trends on magnetic properties of NPs presented in literature are based on homogeneous Fe-oxide nanocrystals. Moving towards hybrid NPs and more complicated nanosystems, leads to even more variables that influence the magnetic properties. This becomes extremely challenging, especially without a reliable basis for comparison with other magnetic nanoparticles presented in literature synthesized by various research groups via different synthetic routes. In any case, the presence of the non-magnetic Pd in the core of Fe-oxide NPs complicates the size-dependence due to variations in the volume of magnetic shell. Therefore, parameters, such as shape, composition, anisotropy, surface functionalization, etc., need to be considered to achieve complete control over the magnetic properties. 

Nevertheless, the reasonably high saturation magnetization values obtained in this study with Pd/Fe-oxide NPs indicate that high-temperature synthesis via thermal decomposition is a suitable method to produce magnetic hybrid NPs. Majority of the hysteresis loops presented by the various batches of Pd/Fe-oxide NPs suggests that the samples are in their superparamagnetic regime and saturation magnetization occurs at relatively low fields. Finally, the best combination of SLP values and *r*_2_-relaxivities in water were obtained for Exp_standard and Exp_10mg. These batches present efficient heating and relaxation time reduction, which encourages further investigations on their application for MR-image guided hyperthermia/thermal ablation and radiotherapy. 

## Figures and Tables

**Table 1:** Summary of the synthetic conditions in seed-mediated synthesis of Pd/Fe-oxide NPs and the resulting average sizes.

Sample	Pd-Seeds (mg)	Heating Rate (°C/min)	[surf]/[Fe] (mol/mol)	Solvent	Average Diameter (nm ± SD)
Exp_standard	20	5	5.5	ODE	19.5 ± 1.5
Exp_10mg	10	5	5.5	ODE	21.0 ± 2.0
Exp_nDS	20	5	5.5	ODE	14.7 ± 1.8
Exp_3 °C	20	3	5.5	ODE	13.3 ± 2.2
Exp_7 °C	20	7	5.5	ODE	18.9 ± 2.1
Exp_[surf]/[Fe] = 2	20	5	2	ODE	9.8 ± 1.1
Exp_[surf]/[Fe] = 3.25	20	5	3.25	ODE	16.1 ± 4.3
Exp_[surf]/[Fe] = 11	20	5	11	ODE	16.3 ± 1.7
Exp_OAm	20	5	5.5 ^a^	ODE	– ^c^
Exp_OA	20	5	5.5 ^b^	ODE	– ^c^
Exp_DPE	20	5	5.5	DPE	12.1 ± 2.1
Exp_ODE:DPE(1:1)	20	5	5.5	ODE:DPE	15.5 ± 1.8
Exp_DBE	20	5	5.5	DBE	17.9 + 2.5

^a^ 2 mL OAm, w/o OA; ^b^ 2 mL OA, w/o OAm; ^c^ not measured; surf = surfactant = [OA] + [OAm]; ODE = 1-octadecene; DPE = diphenyl ether; DBE = dibenzyl ether.

**Table 2:** Dependence of the average diameter of Pd/Fe-oxide NPs on the b.p. of the solvent in which the experiment was conducted.

Solvent	ODE	DBE	ODE:DPE (1:1)	DPE
Boiling point (°C)	315 °C	298 °C	±270 °C	258 °C
Average diameter (nm)	19.5 ± 1.5	17.9 ± 2.5	15.5 + 1.8	12.1 ± 2.1

**Table 3:** Overview of the magnetic properties of Pd/Fe-oxide NPs. Saturation magnetization (M_s_) at 60 kOe, T = 5 K and 300 K, coercivity (H_c_) at T = 5 K and 300 K, and determined blocking temperature (T_B_). Calculated SLP, ILP and *r*_2_ values.

Sample	Saturation Magnetization (M_s_) (emu/g)		Coercivity (H_c_) (Oe)		T_B_ (K)	SLP ^a^ (W/g_Fe_)	ILP (nHm^2^/kg)	*r*_2_ ^b^ (mM^−1^ s^−1^)
	5 K	300 K	5 K	300 K				
Exp_standard	67.9	58.7	20	8	180	113	0.975	220
Exp_flower-like	53.1	45.4	270	31	120	-^c^	-^c^	-^c^
Exp_popcorn-like	58.5	49.0	427	6	>370	-^c^	-^c^	-^c^
Exp_10 mg	69.2	61.4	67	12	200	233	2.011	440
Exp_nDS	62.7	56.3	26	8	100	-^c^	-^c^	-^c^
Exp_3 °C	52.1	45.1	287	8	40	-^c^	-^c^	-^c^
Exp_7 °C	63.7	57.5	213	6	130	102	0.880	90
Exp_[surf]/[Fe] = 2	26.4	20.5	466	10	30	2	0.017	-^c^
Exp_[surf]/[Fe] = 11	62.4	55.8	81	4	120	-^c^	-^c^	-^c^
Exp_DPE	36.9	28.1	236	19	23	-^c^	-^c^	-^c^
Exp_ODE:DPE (1:1)	46.5	39.9	56	9	120	-^c^	-^c^	-^c^
Exp_DBE	63.6	55.4	13	4	160	-^c^	-^c^	-^c^

^a^ Measured at 346 kHz, 23 mT; ^b^ measured at 9.4 T and 25 °C; ^c^ not measured.

**Table 4:** The Mössbauer fitted parameters of Pd/Fe-oxide NPs at 300 K.

Sample	Phase	IS (mm s^−1^)	QS (mm s^−1^)	Hyperfine Field (T)	Γ (mm s^−1^)	Spectral Contribution (%)
Exp_standard	Fe^3+^ (Fe_3_O_4_, A)	0.32	−0.01	47.2	0.46	24
	Fe^2.5+δ^ (Fe_3_O_4_, B)	0.54	−0.01	43.3	1.03	76
Exp_flower-like	Fe^3+^ (Fe_3_O_4_, A)	0.31	−0.02	48.2	0.50	22
	Fe^2.5+δ^ (Fe_3_O_4_, B)	0.60	0.02	44.6	0.93	70
	Fe^3+^ (SPM)	0.19	0.98	-	0.95	8
Exp_10mg	Fe^3+^ (Fe_3_O_4_, A)	0.34	−0.02	47.5	0.57	38
	Fe^2.5+δ^ (Fe_3_O_4_, B)	0.50	−0.01	43.2	1.12	62
Exp_nDS	Fe^3+^ (Fe_3_O_4_, A)	0.33	−0.01	47.8	0.53	41
	Fe^2.5+δ^ (Fe_3_O_4_, B)	0.49	0.02	43.7	0.99	59
Exp_7 °C	Fe^3+^ (Fe_3_O_4_, A)	0.34	−0.02	47.7	0.56	42
	Fe^2.5+δ^ (Fe_3_O_4_, B)	0.46	−0.03	43.6	1.07	58
Exp_[surf]/[Fe] = 2	Fe^3+^ (SPM)	0.33	0.79	-	0.78	100
Exp_ODE:DPE(1:1)	Fe^3+^ (SPM)	0.36	0.80	-	0.74	41
	Fe^3+^ (γ-Fe_2_O_3_)	0.34	−0.02	45.6	0.68	59
Exp_DBE	Fe^3+^ (γ-Fe_2_O_3_)	0.33	−0.01	48.1	0.55	53
	Fe^3+^ (γ-Fe_2_O_3_)	0.36	−0.01	44.0	1.04	47

Experimental uncertainties: Isomer shift: I.S. ± 0.02 mm s^−1^; Quadrupole splitting: Q.S. ± 0.02 mm s^−1^; Line width: Γ ± 0.03 mm s^−1^; Hyperfine field: ±0.1 T; Spectral contribution: ±3%.

## Data Availability

The data used in this study can be accessed at https://doi.org/10.4121/21062629.

## Supplementary Materials

The following supporting information can be downloaded at: https://www.mdpi.com/article/10.3390/nano12203649/s1, Figure S1: Characterization of OAm-capped and nDS-capped Pd-seeds: (A and B) TEM images and (C and D) size distribution; Figure S2: TEM image of Exp_10mg NPs; Figure S3: TEM image of Exp_7 °C, Figure S4: Diameter of Pd/Fe-oxide NPs as function of the ligand/precursor molar ratio; Figure S5: TEM images of NPs prepared with OAm only (A) and with OA only (B); Figure S6: SQUID measurements at T = 5K and coercivity (H_C_) in the inset; Figure S7: Inverse of the *T*_2_-relaxation times as a function of iron concentration for Exp_10mg.

## References

[1] Vangijzegem T., Stanicki D., Laurent S. (2019). Magnetic iron oxide nanoparticles for drug delivery: Applications and characteristics. Expert Opin. Drug Deliv..

[2] Gossuin Y., Gillis P., Hocq A., Vuong Q.L., Roch A. (2009). Magnetic resonance relaxation properties of superparamagnetic particles. WIREs Nanomed. Nanobiotechnol..

[3] Blanco-Andujar C., Walter A., Cotin G., Bordeianu C., Mertz D., Felder-Flesch D., Begin-Colin S. (2016). Design of iron oxide-based nanoparticles for MRI and magnetic hyperthermia. Nanomedicine.

[4] Laurent S., Saei A.A., Behzadi S., Panahifar A., Mahmoudi M. (2014). Superparamagnetic iron oxide nanoparticles for delivery of therapeutic agents: Opportunities and challenges. Expert Opin. Drug Deliv..

[5] Sharma S., Lamichhane N., Parul Sen T., Roy I. (2021). Iron oxide nanoparticles conjugated with organic optical probes for in vivo diagnostic and therapeutic applications. Nanomedicine.

[6] Torres M.d.R.R., Tavare R., Paul R.L., Jauregui-Osoro M., Protti A., Glaria A., Varma G., Szanda I., Blower P.J. (2011). Synthesis of 64Cu(II)-bis(dithiocarbamatebisphosphonate) and its conjugation with superparamagnetic iron oxide nanoparticles: In vivo evaluation as dual-modality PET-MRI agent. Angew. Chem. Int. Ed. Engl..

[7] Sanchez L.M., Alvarez V.A. (2019). Advances in Magnetic Noble Metal/Iron-Based Oxide Hybrid Nanoparticles as Biomedical Devices. Bioengineering.

[8] Boros E., Bowen A.M., Josephson L., Vasdev N., Holland J.P. (2015). Chelate-free metal ion binding and heat-induced radiolabeling of iron oxide nanoparticles. Chem. Sci..

[9] Ma D., Mohapatra S., Nguyen T.A., Nguyen-Tri P. (2019). Chapter 1—Hybrid Nanoparticles: An Introduction. Noble Metal-Metal Oxide Hybrid Nanoparticles.

[10] Wu S., Sun A., Zhai F., Wang J., Xu W., Zhang Q., Volinsky A.A. (2011). Fe_3_O_4_ magnetic nanoparticles synthesis from tailings by ultrasonic chemical co-precipitation. Mater. Lett..

[11] Qi H., Yan B., Li C., Lu W. (2011). Synthesis and characterization of water-soluble magnetite nanocrystals via one-step sol-gel pathway. Sci. China Phys. Mech. Astron..

[12] Jarzyna P.A., Skajaa T., Gianella A., Cormode D.P., Samber D.D., Dickson S.D., Chen W., Griffioen A.W., Fayad Z.A., Mulder W.J.M. (2009). Iron oxide core oil-in-water emulsions as a multifunctional nanoparticle platform for tumor targeting and imaging. Biomaterials.

[13] Cotin G., Perton F., Blanco-Andujar C., Pichon B., Mertz D., Bégin-Colin S., Fratila R.M., De La Fuente J.M. (2019). Chapter 2—Design of Anisotropic Iron-Oxide-Based Nanoparticles for Magnetic Hyperthermia. Nanomaterials for Magnetic and Optical Hyperthermia Applications.

[14] Chai Y., Feng F., Li Q., Yu C., Feng X., Lu P., Yu X., Ge M., Wang X., Yao L. (2019). One-Pot Synthesis of High-Quality Bimagnetic Core/Shell Nanocrystals with Diverse Exchange Coupling. J. Am. Chem. Soc..

[15] Xia Y., Gilroy K.D., Peng H.-C., Xia X. (2017). Seed-Mediated Growth of Colloidal Metal Nanocrystals. Angew. Chem. Int. Ed..

[16] Tancredi P., da Costa L.S., Calderon S., Moscoso-Londoño O., Socolovsky L.M., Ferreira P.J., Muraca D., Zanchet D., Knobel M. (2019). Exploring the synthesis conditions to control the morphology of gold-iron oxide heterostructures. Nano Res..

[17] Carbone L., Cozzoli P.D. (2010). Colloidal heterostructured nanocrystals: Synthesis and growth mechanisms. Nano Today.

[18] Fantechi E., Roca A.G., Sepúlveda B., Torruella P., Estradé S., Peiró F., Coy E., Jurga S., Bastús N.G., Nogués J. (2017). Seeded Growth Synthesis of Au–Fe_3_O_4_ Heterostructured Nanocrystals: Rational Design and Mechanistic Insights. Chem. Mater..

[19] Wei Y., Klajn R., Pinchuk A.O., Grzybowski B.A. (2008). Synthesis, Shape Control, and Optical Properties of Hybrid Au/Fe_3_O_4_ “Nanoflowers”. Small.

[20] Jang Y., Chung J., Kim S., Jun S.W., Kim B.H., Lee D.W., Kim B.M., Hyeon T. (2011). Simple synthesis of Pd–Fe_3_O_4_ heterodimer nanocrystals and their application as a magnetically recyclable catalyst for Suzuki cross-coupling reactions. Phys. Chem. Chem. Phys..

[21] Lee J., Chung J., Byun S.M., Kim B.M., Lee C. (2013). Direct catalytic C–H arylation of imidazo[1,2-a]pyridine with aryl bromides using magnetically recyclable Pd–Fe_3_O_4_ nanoparticles. Tetrahedron.

[22] Jang S., Hira S.A., Annas D., Song S., Yusuf M., Park J.C., Park S., Park K.H. (2019). Recent Novel Hybrid Pd–Fe_3_O_4_ Nanoparticles as Catalysts for Various C–C Coupling Reactions. Processes.

[23] Lee W.-S., Byun S., Kwon J., Kim B.M. (2016). Magnetic Pd–Fe_3_O_4_ Heterodimer Nanocrystals as Recoverable Catalysts for Ligand-Free Hiyama Cross-Coupling Reactions. Bull. Korean Chem. Soc..

[24] Yu Y., Anderson L.L., Li Z., Mellenberg D.E., Nath R., Schell M.C., Waterman F.M., Wu A., Blasko J.C. (1999). Permanent prostate seed implant brachytherapy: Report of the American Association of Physicists in Medicine Task Group No. 64. Med. Phys..

[25] Sharkey J., Cantor A., Solc Z., Huff W., Chovnick S.D., Behar R.J., Perez R., Otheguy J., Rabinowitz R. (2005). 103Pd brachytherapy versus radical prostatectomy in patients with clinically localized prostate cancer: A 12-year experience from a single group practice. Brachytherapy.

[26] Kainz K. (2006). Radiation Oncology Physics: A Handbook for Teachers and Students. Med. Phys..

[27] Maier A., Djanashvili K., Denkova A.G., van Rhoon G.C., Pignol J.-P., van Oossanen R. (2021). Synthesis of Hybride Palladium(103)/Iron Oxide Nanoparticles for Thermobrachy Therapy. Eur. Pat. Appl..

[28] Chen S., Si R., Taylor E., Janzen J., Chen J. (2012). Synthesis of Pd/Fe_3_O_4_ Hybrid Nanocatalysts with Controllable Interface and Enhanced Catalytic Activities for CO Oxidation. J. Phys. Chem. C.

[29] Lin F.-H., Chen W., Liao Y.-H., Doong R.-A., Li Y. (2011). Effective approach for the synthesis of monodisperse magnetic nanocrystals and M-Fe_3_O_4_ (M = Ag, Au, Pt, Pd) heterostructures. Nano Res..

[30] Mazumder V., Sun S. (2009). Oleylamine-Mediated Synthesis of Pd Nanoparticles for Catalytic Formic Acid Oxidation. J. Am. Chem. Soc..

[31] Ganesan M., Freemantle R.G., Obare S.O. (2007). Monodisperse Thioether-Stabilized Palladium Nanoparticles:  Synthesis, Characterization, and Reactivity. Chem. Mater..

[32] Liu S., Guo S., Sun S., You X.-Z. (2015). Dumbbell-like Au-Fe_3_O_4_ nanoparticles: A new nanostructure for supercapacitors. Nanoscale.

[33] Jin Y., Jia C., Huang S.-W., O’Donnell M., Gao X. (2010). Multifunctional nanoparticles as coupled contrast agents. Nat. Commun..

[34] Klencsár Z. (1997). Mössbauer spectrum analysis by Evolution Algorithm. Nucl. Instrum. Methods Phys. Res. Sect. B Beam Interact. Mater. At..

[35] Wang C., Yin H., Dai S., Sun S. (2010). A General Approach to Noble Metal−Metal Oxide Dumbbell Nanoparticles and Their Catalytic Application for CO Oxidation. Chem. Mater..

[36] Lassenberger A., Grünewald T.A., van Oostrum P.D.J., Rennhofer H., Amenitsch H., Zirbs R., Lichtenegger H.C., Reimhult E. (2017). Monodisperse Iron Oxide Nanoparticles by Thermal Decomposition: Elucidating Particle Formation by Second-Resolved in Situ Small-Angle X-ray Scattering. Chem. Mater..

[37] Baaziz W., Pichon B.P., Fleutot S., Liu Y., Lefevre C., Greneche J.-M., Toumi M., Mhiri T., Begin-Colin S. (2014). Magnetic Iron Oxide Nanoparticles: Reproducible Tuning of the Size and Nanosized-Dependent Composition, Defects, and Spin Canting. J. Phys. Chem. C.

[38] Maity D., Choo S.-G., Yi J., Ding J., Xue J.M. (2009). Synthesis of magnetite nanoparticles via a solvent-free thermal decomposition route. J. Magn. Magn. Mater..

[39] Sun S., Zeng H., Robinson D.B., Raoux S., Rice P.M., Wang S.X., Li G. (2004). Monodisperse MFe_2_O_4_ (M = Fe, Co, Mn) Nanoparticles. J. Am. Chem. Soc..

[40] Xie J., Peng S., Brower N., Pourmand N., Wang S.X., Sun S. (2006). One-pot synthesis of monodisperse iron oxide nanoparticles for potential biomedical applications. Pure Appl. Chem..

[41] Yu H., Chen M., Rice P.M., Wang S.X., White R.L., Sun S. (2005). Dumbbell-like bifunctional Au-Fe_3_O_4_ nanoparticles. Nano Lett..

[42] Efremova M.V., Naumenko V.A., Spasova M., Garanina A.S., Abakumov M.A., Blokhina A.D., Melnikov P.A., Prelovskaya A.O., Heidelmann M., Li Z.-A. (2018). Magnetite-Gold nanohybrids as ideal all-in-one platforms for theranostics. Sci. Rep..

[43] Han D.H., Wang J.P., Luo H.L. (1994). Crystallite size effect on saturation magnetization of fine ferrimagnetic particles. J. Magn. Magn. Mater..

[44] Vargas J.M., Lawton J., Vargas N.M., Schuller I.K., Sowko N.J., Huang M.-X., Zhang M. (2020). Temperature trends and correlation between SQUID superparamagnetic relaxometry and dc-magnetization on model iron-oxide nanoparticles. J. Appl. Phys..

[45] Skumryev V., Stoyanov S., Zhang Y., Hadjipanayis G., Givord D., Nogués J. (2003). Beating the superparamagnetic limit with exchange bias. Nature.

[46] Kolhatkar A.G., Jamison A.C., Litvinov D., Willson R.C., Lee T.R. (2013). Tuning the magnetic properties of nanoparticles. Int. J. Mol. Sci..

[47] Saragi T., Sinaga H.D., Rahmi F., Pramesti G.A., Sugiarto A., Therigan A., Syakir N., Hidayat S., Risdiana R. (2020). Blocking Temperature of Magnetite Nanoparticles Fe_3_O_4_ Encapsulated Silicon Dioxide SiO_2_. Key Eng. Mater..

[48] Majetich S.A., Coey M., Parkin S. (2020). Magnetic Nanoparticles. Handbook of Magnetism and Magnetic Materials.

[49] Mohapatra J., Mitra A., Bahadur D., Aslam M. (2012). Surface controlled fabrication of MFe_2_O_4_ (M = Mn, Fe, Co, Ni and Zn) nanoparticles and their magnetic properties. Cryst. Eng. Comm..

[50] Nedelkoski Z., Kepaptsoglou D., Lari L., Wen T., Booth R.A., Oberdick S.D., Galindo P.L., Ramasse Q.M., Evans R.F.L., Majetich S. (2017). Origin of reduced magnetization and domain formation in small magnetite nanoparticles. Sci. Rep..

[51] Guardia P., Labarta A., Batlle X. (2011). Tuning the Size, the Shape, and the Magnetic Properties of Iron Oxide Nanoparticles. J. Phys. Chem. C.

[52] Mornet S., Vasseur S., Grasset F., Duguet E. (2004). Magnetic nanoparticle design for medical diagnosis and therapy. J. Mater. Chem..

[53] Gazeau F., Levy M., Wilhelm C. (2008). Optimizing magnetic nanoparticle design for nanothermotherapy. Nanomedicine.

[54] Natividad E., Castro M., Mediano A. (2009). Adiabatic vs. non-adiabatic determination of specific absorption rate of ferrofluids. J. Magn. Magn. Mater..

[55] Wildeboer R.R., Southern P., Pankhurst Q.A. (2014). On the reliable measurement of specific absorption rates and intrinsic loss parameters in magnetic hyperthermia materials. J. Phys. D Appl. Phys..

[56] Jeun M., Lee S., Kyeong Kang J., Tomitaka A., Wook Kang K., Il Kim Y., Takemura Y., Chung K.-W., Kwak J., Bae S. (2012). Physical limits of pure superparamagnetic Fe_3_O_4_ nanoparticles for a local hyperthermia agent in nanomedicine. Appl. Phys. Lett..

[57] Filippousi M., Angelakeris M., Katsikini M., Paloura E., Efthimiopoulos I., Wang Y., Zamboulis D., Van Tendeloo G. (2014). Surfactant Effects on the Structural and Magnetic Properties of Iron Oxide Nanoparticles. J. Phys. Chem. C.

[58] Wells J., Ortega D., Steinhoff U., Dutz S., Garaio E., Sandre O., Natividad E., Cruz M.M., Brero F., Southern P. (2021). Challenges and recommendations for magnetic hyperthermia characterization measurements. Int. J. Hyperth..

[59] Efremova M.A.-O., Nalench Y.A.-O., Myrovali E., Garanina A.S., Grebennikov I.S., Gifer P.K., Abakumov M.A., Spasova M., Angelakeris M., Savchenko A.G. (2018). Size-selected Fe(3)O(4)-Au hybrid nanoparticles for improved magnetism-based theranostics. Beilstein J. Nanotechnol..

[60] Ganapathe L.S., Mohamed M.A., Mohamad Yunus R., Berhanuddin D.D. (2020). Magnetite (Fe_3_O_4_) Nanoparticles in Biomedical Application: From Synthesis to Surface Functionalisation. Magnetochemistry.

[61] Jeon M., Halbert M.V., Stephen Z.R., Zhang M. (2021). Iron Oxide Nanoparticles as T1 Contrast Agents for Magnetic Resonance Imaging: Fundamentals, Challenges, Applications, and Prospectives. Adv. Mater..

[62] Laurent S., Forge D., Port M., Roch A., Robic C., Vander Elst L., Muller R.N. (2008). Magnetic Iron Oxide Nanoparticles: Synthesis, Stabilization, Vectorization, Physicochemical Characterizations, and Biological Applications. Chem. Rev..

