# Supplementary material for: From Structure to Function: Understanding Synthetic Conditions in Relation to Magnetic Properties of Hybrid Pd/Fe-Oxide Nanoparticles
Source: Nanomaterials (Basel). 2022 Oct 18;12(20):3649. doi: 10.3390/nano12203649 (PMC9612236; doi:10.3390/nano12203649)
Supplement: Supplementary file 1 [file nanomaterials-12-03649-s001.zip › nanomaterials-1933952-supplementary.pdf]

Supplementary Material

From Structure to Function: Understanding Synthetic Conditions in Relation to Magnetic Properties of Hybrid Pd/Fe-Oxide Nanoparticles

Alexandra Maier [1], Rogier van Oossanen [2,3], Gerard C. van Rhoon [2,3], Jean-Philippe Pignol [4], Iulian Dugulan [3], Antonia G. Denkova [3] and Kristina Djanashvili [1,*]

[1] Department of Biotechnology, Delft University of Technology, Van Der Maasweg 9, 2629 HZ Delft, The Netherlands
[2] Department of Radiotherapy, Erasmus MC Cancer Institute, University Medical Center, 3008 AE Rotterdam, The Netherlands
[3] Department of Radiation Science and Technology, Delft University of Technology, Mekelweg 15, 2629 JB Delft, The Netherlands
[4] Department of Physics and Atmospheric Sciences, Dalhousie University, Sir James Dunn Bldg., Halifax, NS B3H 4J5, Canada
* Correspondence: k.djanashvili@tudelft.nl; Tel.: +31-15-27-85092

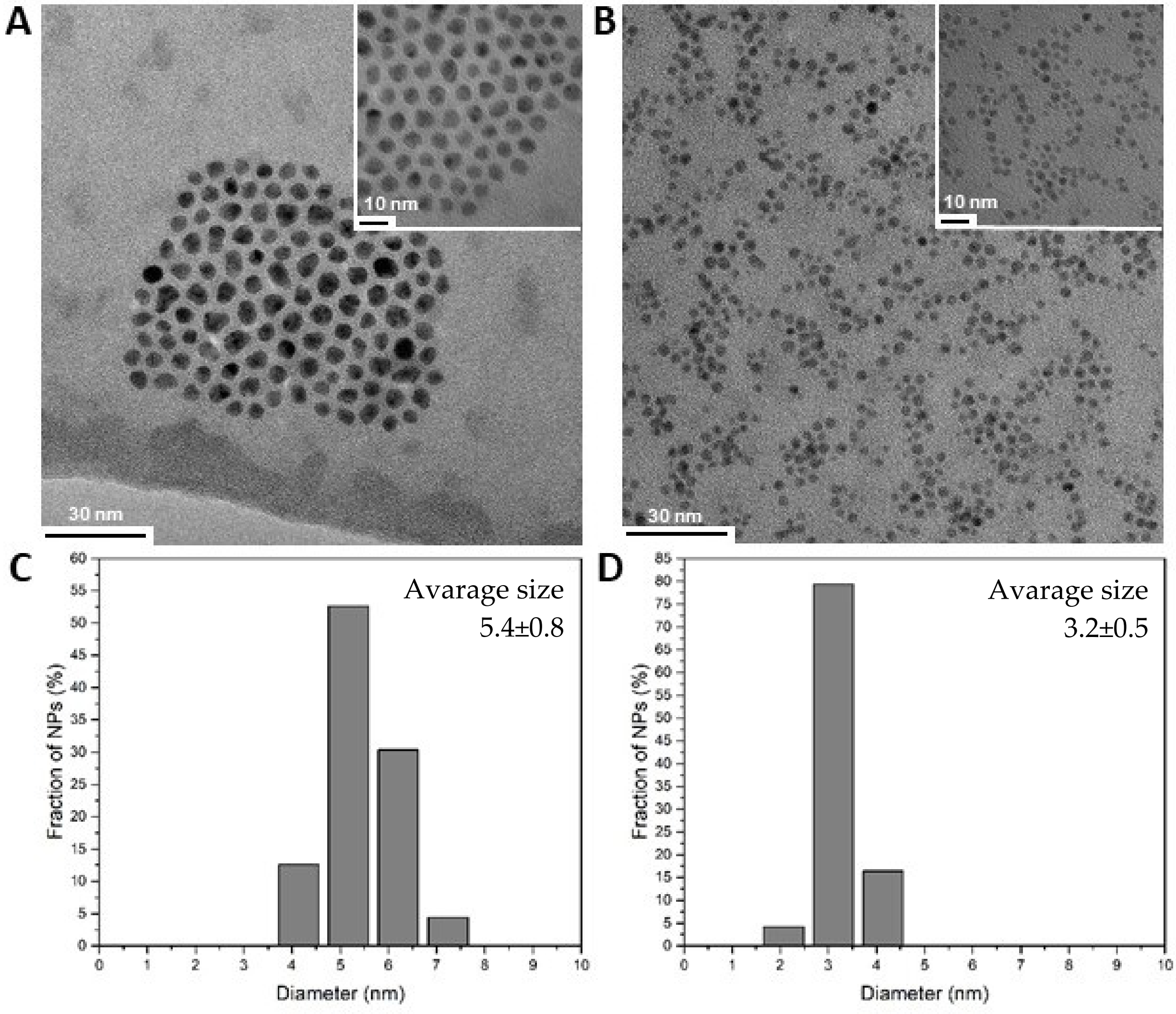

Figure S1. Characterization of OAm-capped (left) and nDS-capped (right) Pd-seeds: (**A**,**B**) TEM images and (**C**,**D**) size distributions.

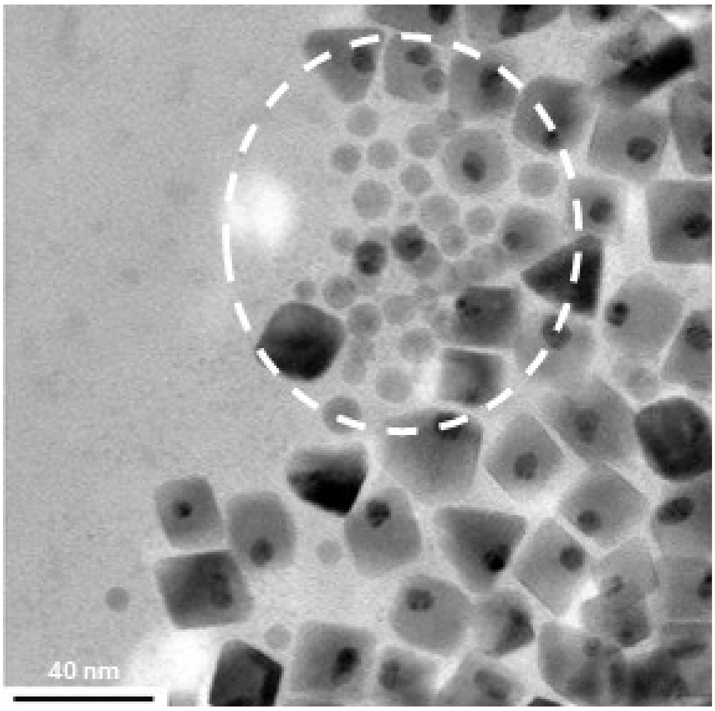

Figure S2. Second population of small iron oxide nanocrystals found in Exp_10mg.

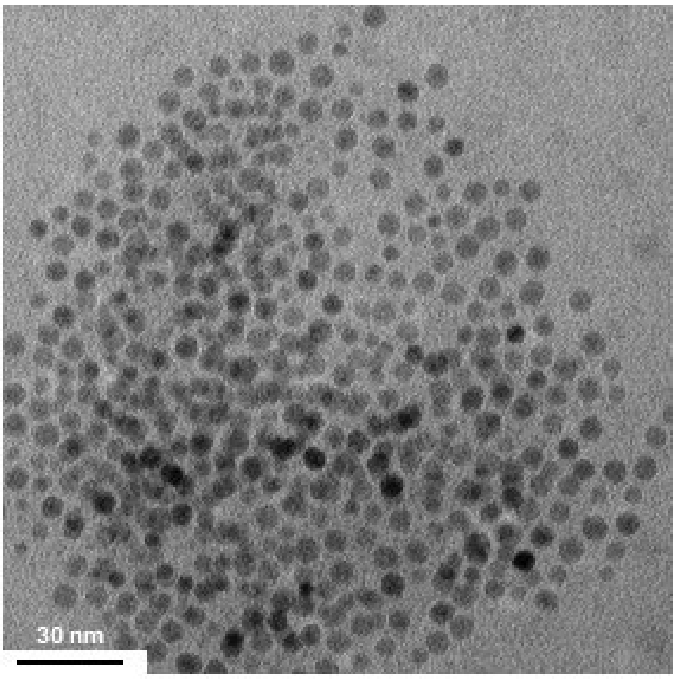

Figure S3. Homogeneous nucleation in the form of Fe-oxide NPs found in Exp_7 °C.

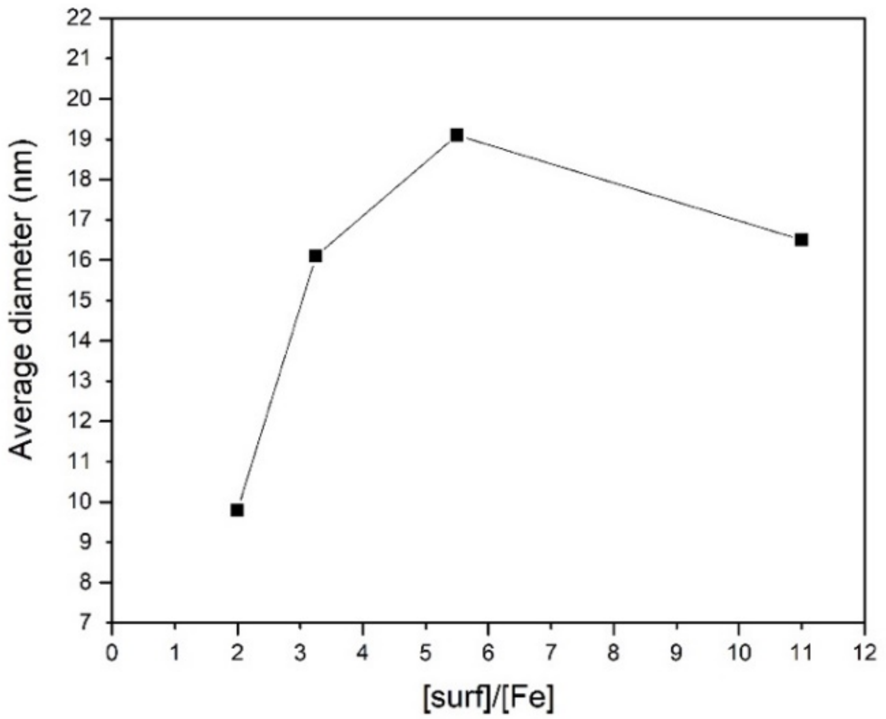

Figure S4. Average diameter of the Pd/Fe-oxide hybrid NPs as a function of the ligand/precursor molar ratio.

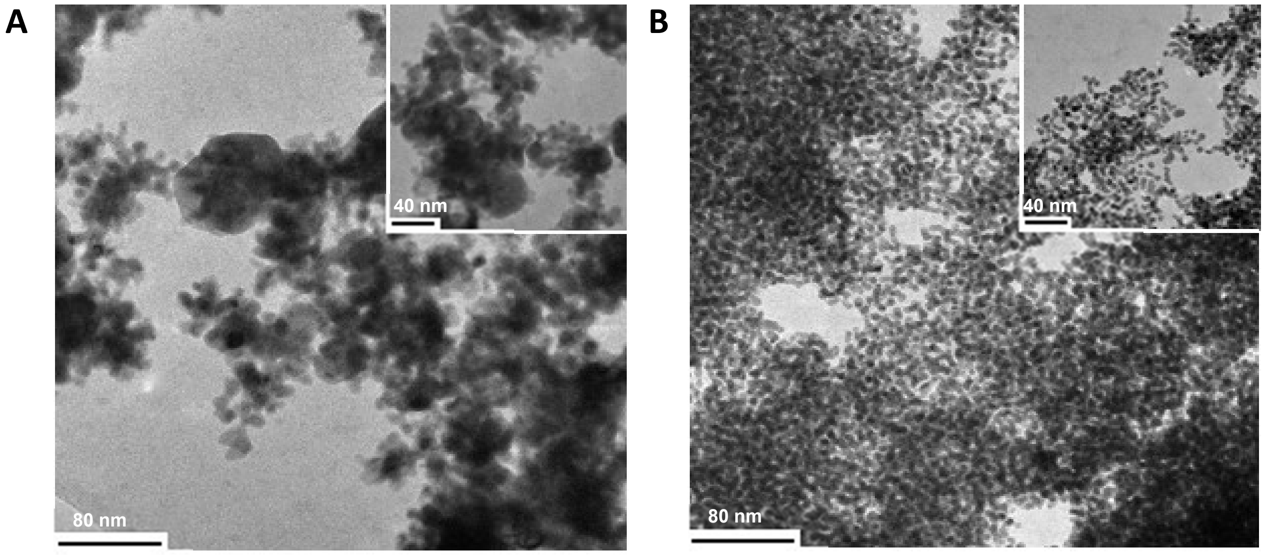

Figure S5. TEM images of NPs prepared with (**A**) only OAm (Exp_OAm) and (**B**) only OA (Exp_OA).

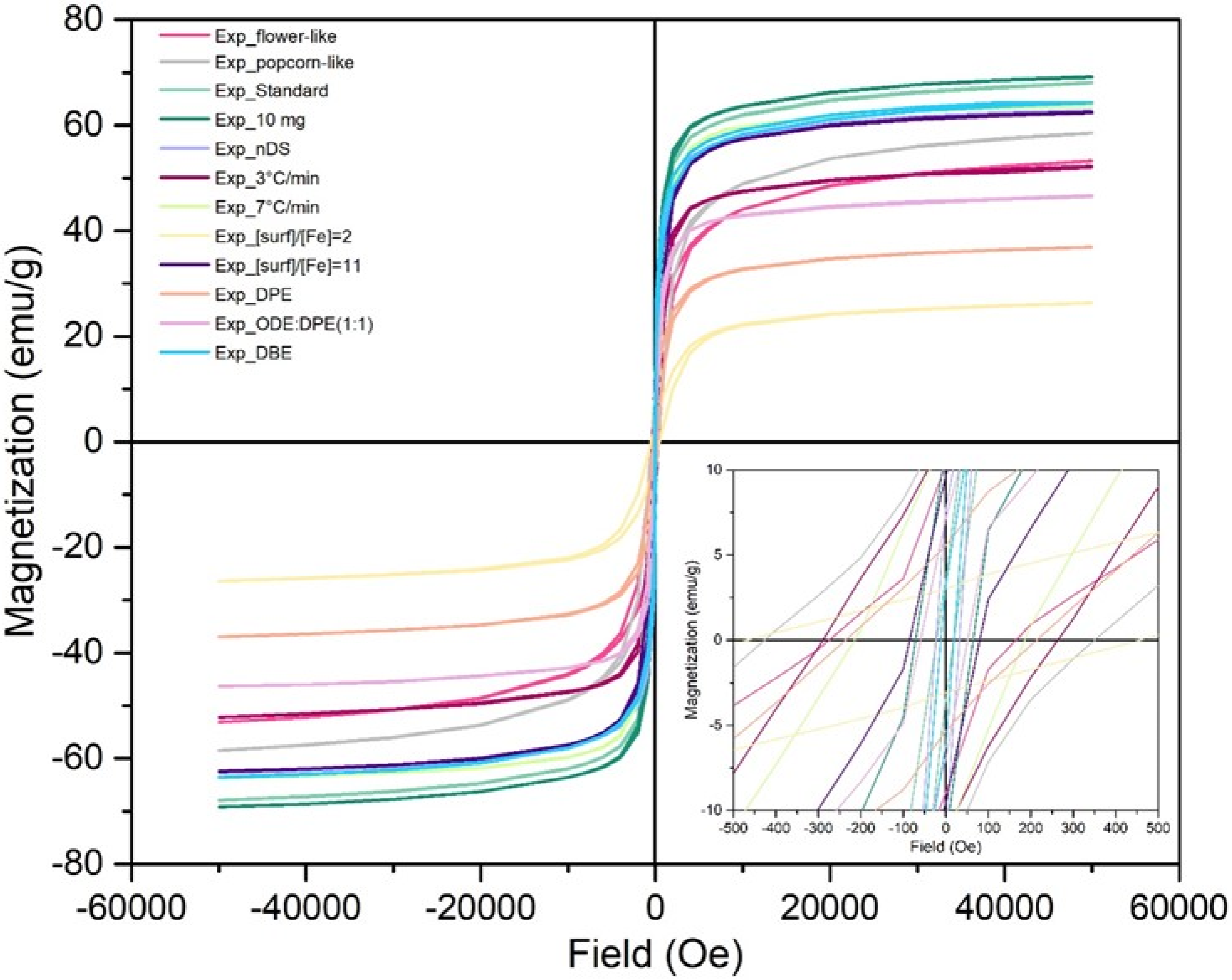

Figure S6. SQUID measurements at T = 5 K and coercivity (H_c) in the inset.

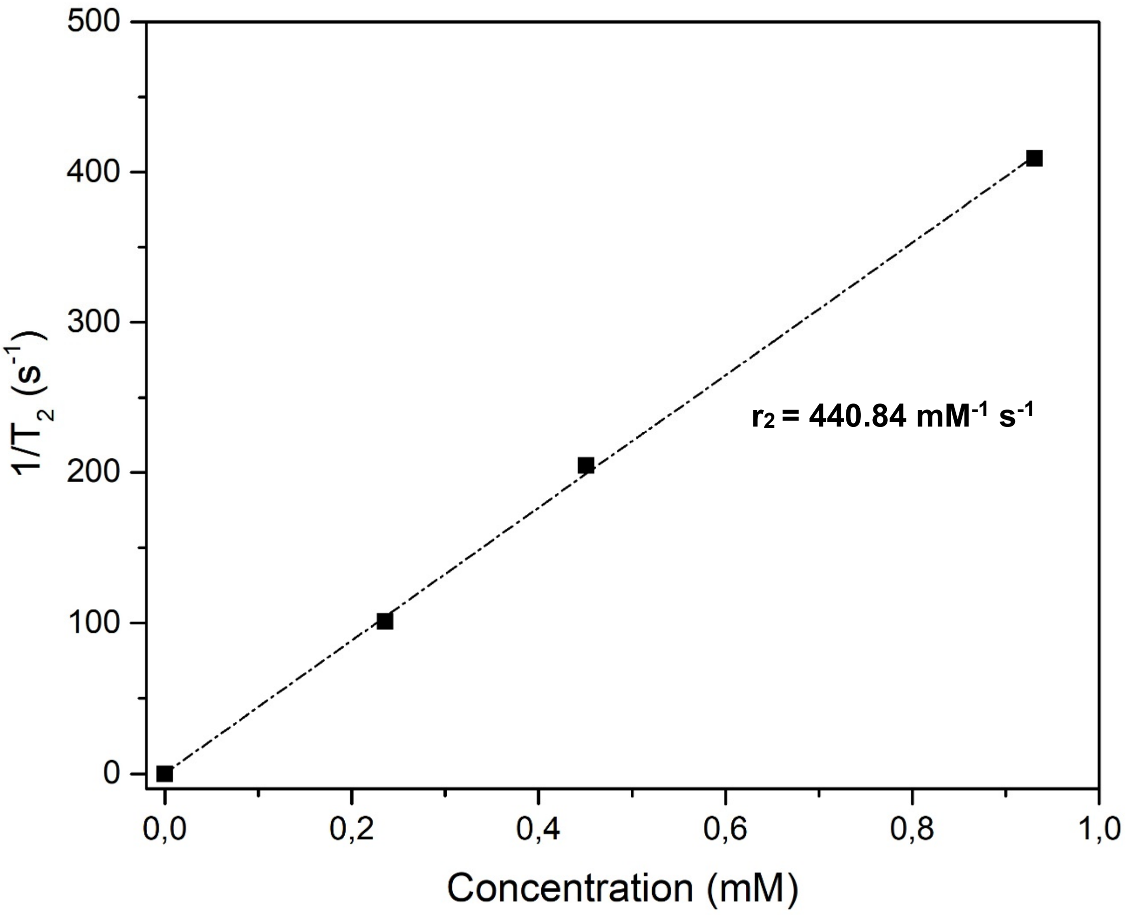

Figure S7. Inverse of the T_2-relaxation times as a function of iron concentration (determined by ICP-OES) for Exp_10mg.
